# TREM2 activation alleviates neural damage via Akt/CREB/BDNF signalling after traumatic brain injury in mice

**DOI:** 10.1186/s12974-022-02651-3

**Published:** 2022-12-03

**Authors:** Jin Yan, Yuan Zhang, Lin Wang, Zhao Li, Shuang Tang, Yingwen Wang, Nina Gu, Xiaochuan Sun, Lin Li

**Affiliations:** 1 Department of Neurosurgery, The First Affiliated Hospital of Chongqing Medical University, 1 Youyi Rd, Chongqing, 400016 China; 2 Department of Neurosurgery, Nanchong Central Hospital, The Second Clinical Medical College of North Sichuan Medical College, Nanchong, China; 3 Department of Neurosurgery, Chengdu Integrated TCM & Western Medicine Hospital, Chengdu, China; 4 Department of Neurosurgery, Suining Central Hospital, Suining, China; 5 Department of Neuro-oncology, Chongqing University Cancer Hospital, Chongqing, China; 6 Department of Neurosurgery, The Affiliated Hospital of North Sichuan Medical College, Nanchong, China

**Keywords:** Traumatic brain injury, TREM2, Neuroinflammation, Neural apoptosis, Cognitive deficits

## Abstract

**Background:**

Neuroinflammation is one of the most important processes in secondary injury after traumatic brain injury (TBI). Triggering receptor expressed on myeloid cells 2 (TREM2) has been proven to exert neuroprotective effects in neurodegenerative diseases and stroke by modulating neuroinflammation, and promoting phagocytosis and cell survival. However, the role of TREM2 in TBI has not yet been elucidated. In this study, we are the first to use COG1410, an agonist of TREM2, to assess the effects of TREM2 activation in a murine TBI model.

**Methods:**

Adult male wild-type (WT) C57BL/6 mice and adult male TREM2 KO mice were subjected to different treatments. TBI was established by the controlled cortical impact (CCI) method. COG1410 was delivered 1 h after CCI via tail vein injection. Western blot analysis, immunofluorescence, laser speckle contrast imaging (LSCI), neurological behaviour tests, brain electrophysiological monitoring, Evans blue assays, magnetic resonance imaging (MRI), and brain water content measurement were performed in this study.

**Results:**

The expression of endogenous TREM2 peaked at 3 d after CCI, and it was mainly expressed on microglia and neurons. We found that COG1410 improved neurological functions within 3 d, as well as neurological functions and brain electrophysiological activity at 2 weeks after CCI. COG1410 exerted neuroprotective effects by inhibiting neutrophil infiltration and microglial activation, and suppressing neuroinflammation after CCI. In addition, COG1410 treatment alleviated blood brain barrier (BBB) disruption and brain oedema; furthermore, COG1410 promoted cerebral blood flow (CBF) recovery at traumatic injury sites after CCI. In addition, COG1410 suppressed neural apoptosis at 3 d after CCI. TREM2 activation upregulated p-Akt, p-CREB, BDNF, and Bcl-2 and suppressed TNF-α, IL-1β, Bax, and cleaved caspase-3 at 3 d after CCI. Moreover, TREM2 knockout abolished the effects of COG1410 on vascular phenotypes and microglial states. Finally, the neuroprotective effects of COG1410 were suppressed by TREM2 depletion.

**Conclusions:**

Altogether, we are the first to demonstrate that TREM2 activation by COG1410 alleviated neural damage through activation of Akt/CREB/BDNF signalling axis in microglia after CCI. Finally, COG1410 treatment improved neurological behaviour and brain electrophysiological activity after CCI.

**Graphical Abstract:**

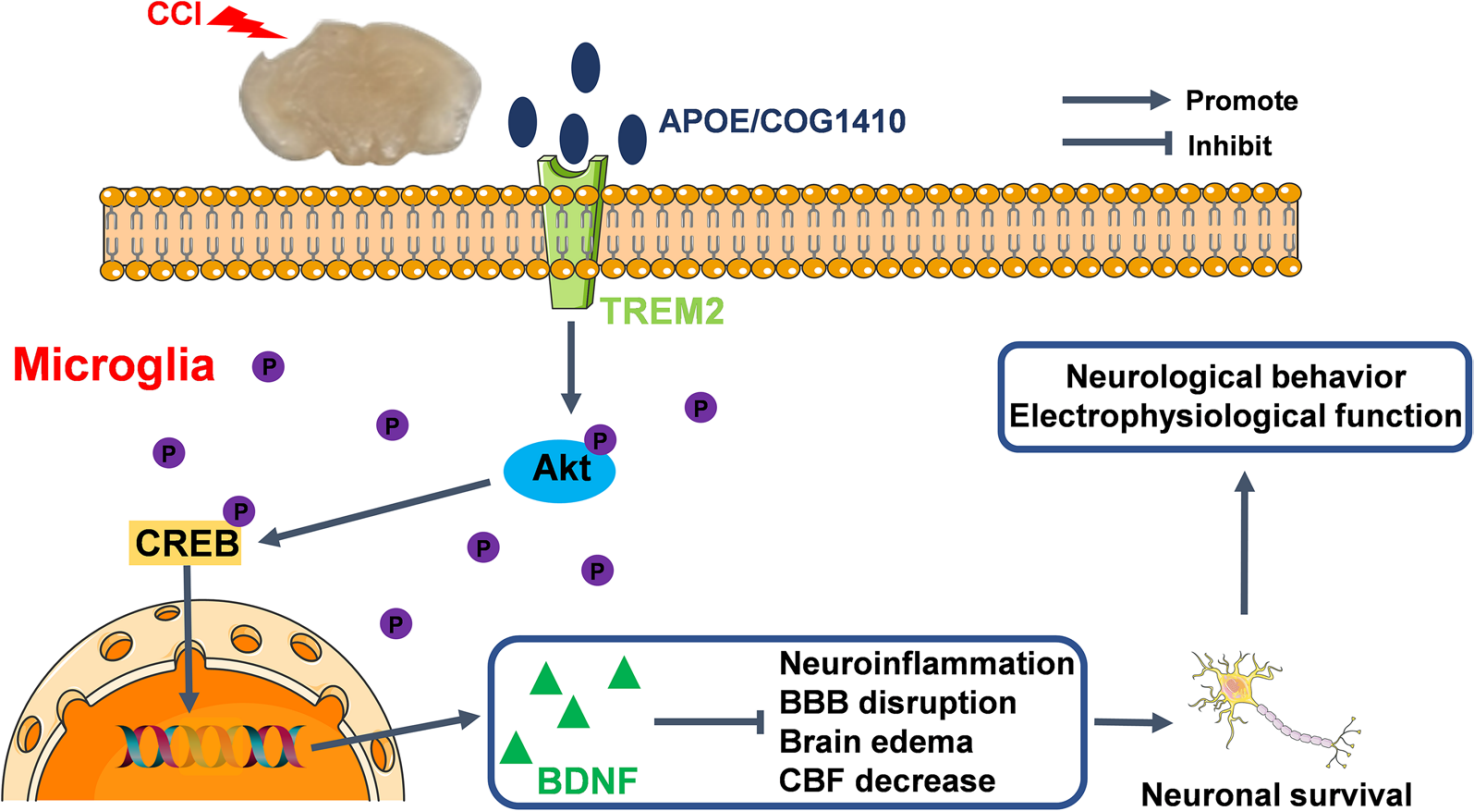

**Supplementary Information:**

The online version contains supplementary material available at 10.1186/s12974-022-02651-3.

## Introduction

Traumatic brain injury (TBI), one of the leading causes of mortality and disability in China, causes a considerable socioeconomic burden. In addition, China has more TBI patients than most other countries worldwide, making this condition a major public health concern in the Chinese society [1, 2]. Therefore, it is of great significance for us to find potential therapeutic targets for TBI.

The treatment of TBI is particularly challenging because it is heterogeneous in nature and often induces complex pathogenesis pathways. There is an ordered temporal and spatial pathological evolution after TBI, i.e. primary and secondary brain injury [3]. Primary injury occurs at the time of TBI onset and is deteriorated by acute systemic complications, such as hypotension, hypoxia and haemorrhage. Subsequently, the initial mechanical effects of injury lead to various biochemical cascades, which are collectively referred to as “secondary injury” [3, 4]. Secondary injury mainly includes: (1) excitotoxicity induced by excessive glutamate release [5], (2) free radical generation [6], and (3) neuroinflammatory response [7]. Immune responses after TBI are pivotal to the clearance of tissue debris and neural repair and regeneration. However, uncontrolled neuroinflammation can cause additional brain injury [8]. After TBI, cellular membrane disruption causes the release of damage-associated molecular patterns (DAMPs), which are capable of triggering and amplifying the neuroinflammatory response [9]. Subsequently, tumour necrosis factor (TNF)α, and interleukin (IL)-1β are immediately upregulated and drive neuroinflammation [10]. Exorbitant neuroinflammation damages blood brain barrier (BBB) integrity, aggravates brain oedema, and promotes peripheral immune cell infiltration, and neural apoptosis [11–13]. Conversely, these events further amplify neuroinflammation [14–16]. To date, abundant research has focused on the neuroinflammatory response after TBI both in animal models and patients. Some preclinical studies reported that the alleviation of neuroinflammation showed neuroprotective effects in a TBI model [8, 17, 18]. Thus, neuroinflammation is still a promising target for the treatment of neural injury after TBI.

Triggering receptor expressed on myeloid cells 2 (TREM2) is a transmembrane receptor of the immunoglobulin superfamily and is mainly expressed on myeloid cells, such as microglia in the central nervous system [19]. Activation of TREM2 can regulate proliferation, suppress inflammatory cytokine production, promote cell survival, and benefit phagocytosis of apoptotic neurons [19, 20]. To date, increasing evidence has shown the neuroprotective effects of TREM2 in Alzheimer’s disease, multiple sclerosis and Parkinson’s disease [21–23]. In Alzheimer’s disease, a decrease in TREM2 accelerates the process of ageing and neural loss, finally, it reduces microglial activity and leads to neuroinflammation [21]. Kazuya Takahashi et al. reported that intravenous application of TREM2-transduced bone marrow-derived myeloid precursor cells after multiple sclerosis in mice increased lysosomal and phagocytic activity, cleared degenerated myelin, and reduced the inflammatory response at spinal cord lesions [22]. In a Parkinson’s disease animal model, TREM2 deficiency aggravated α-synuclein-induced neurodegeneration and neuroinflammation [23]. The ligands of TREM2 include bacterial products, DNA, lipoproteins, and phospholipids [24]. Recent studies have shown that apolipoprotein E (apoE) is a novel and high-affinity ligand of TREM2 [25, 26]. In a previous study, Chen et al. used an apoE mimic peptide, COG1410, to estimate whether TREM2 activation could attenuate neural damage after experimental intracerebral haemorrhage (ICH). They found that COG1410 treatment improves both short-term and long-term neurological functions by attenuating neuroinflammation and neural apoptosis after ICH. The neuroprotective effects of COG1410 after ICH were mediated by the PI3K/Akt signalling pathway [27]. In addition, TREM2 also showed an antineuroinflammatory effect in experimental ischaemic stroke and subarachnoid haemorrhage [28, 29]. However, whether TREM2 also has neuroprotective effects after TBI has not yet been elucidated. The protein kinase B (Akt)/cAMP-responsive element-binding protein (CREB)/brain-derived neurotrophic factor (BDNF) axis has been proven to have neuroprotective effects against neurodegeneration by preventing neuroinflammation, oxidative stress, apoptosis, and mitochondrial dysfunction [30]. In addition, this signalling pathway can alleviate critical illness-related corticosteroid insufficiency after TBI by promoting neuronal survival [31]. Intriguingly, Akt is a downstream target of TREM2 [27, 32, 33]. Consequently, TREM2 and its downstream Akt/CREB/BDNF signalling pathway may exert neuroprotective effects after TBI. Therefore, studying the underlying mechanisms of TREM2 activity may help to better understand the pathophysiological processes of TBI and may provide novel therapeutic approaches.

In the present study, controlled cortical impact (CCI), one of the most applied TBI models in animal research, was induced in mice to mimic clinical TBI [34]. Inspired by Chen et al., COG1410, an apoE-mimetic peptide, was used to activate TREM2 after CCI. We assumed that TREM2 activation could alleviate neural damage by modulating the Akt/CREB/BDNF signalling axis after CCI.

## Materials and methods

All animals were randomized to the different groups by Microsoft Excel software, and data were collected and analysed in a blinded way. For each animal, different investigators were involved in different stages of the experiments. All procedures on animals were approved by the Ethics Committee of Chongqing Medical University and carried out in accordance with ARRIVE guidelines and the National Institutes of Health Guide for the Care and Use of Laboratory Animals [35].

### Animals

**Fig.1:**
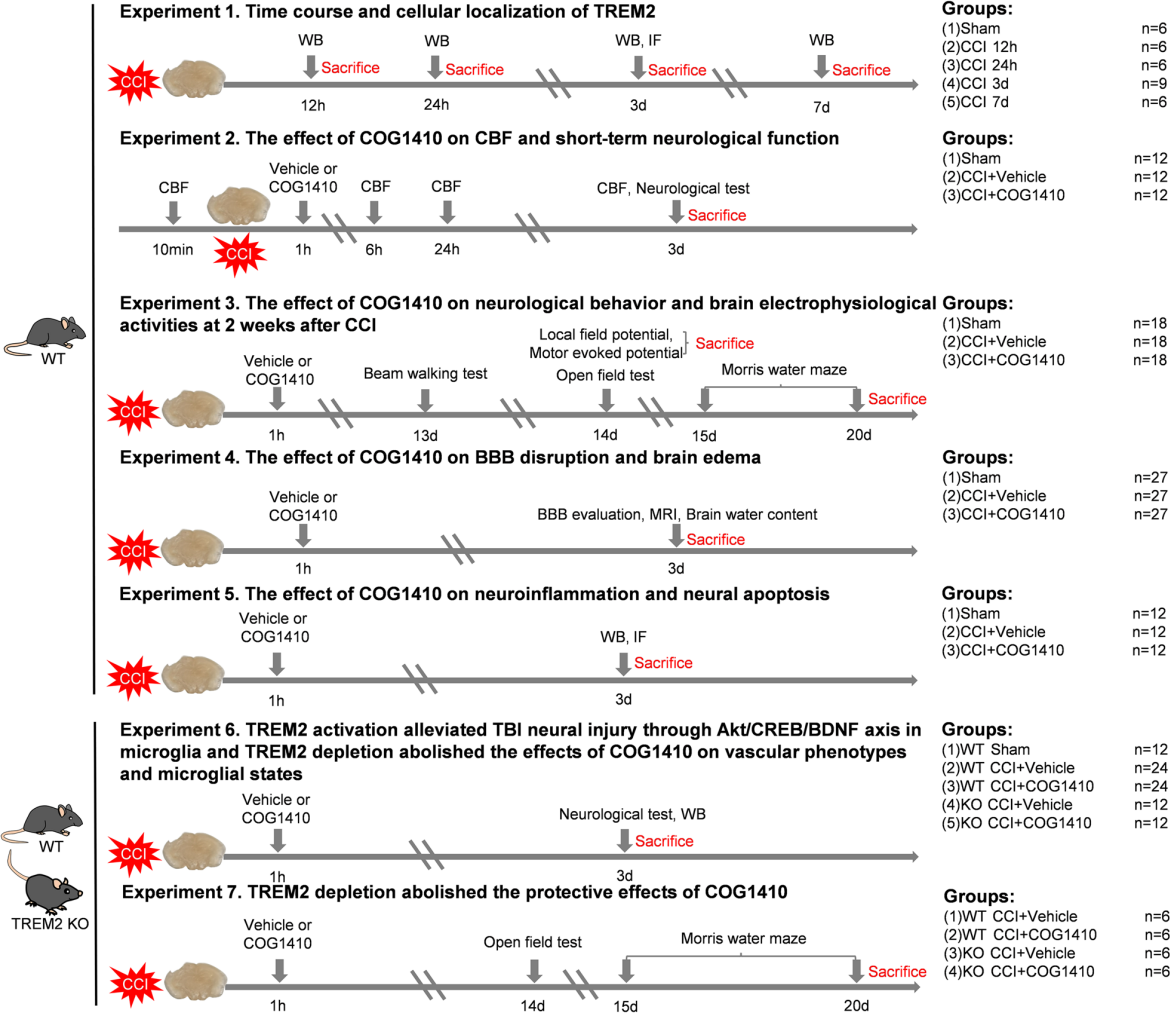
Schematic diagram of different experimental protocols and setups of this study. CCI, controlled cortical impact; TREM2, triggering receptor expressed on myeloid cells 2; WB, Western blot; IF, immunofluorescence; CBF, cerebral blood flow; BBB, blood brain barrier; MRI, magnetic resonance imaging

Adult male wild-type (WT) C57BL/6 mice (8–12 weeks old, 22–30 g) were purchased from the Laboratory Animal Center of Chongqing Medical University (Chongqing, China). Adult male TREM2 KO mice (8–12 weeks old, 22–30 g) were purchased from Jackson Lab (Jackson Labs stock #027197). The TREM2 genotype identification methods and results are shown in the Additional file 1: Additional methods and Fig. S1. All mice were maintained under optimal conditions for hygiene, temperature, and light (12 L:12 D) and allowed food and water ad libitum. All procedures were performed in a specific pathogen-free (SPF) environment, and all tools and materials were sterilized with 75% alcohol. Mice were anaesthetized with isoflurane (3% induction, 2% maintenance) when they underwent any surgeries. At the end of each experiment, mice were killed under deep anaesthesia by pentobarbital sodium (0.3%, 40 mg/kg) and the time points for killing are shown in Fig. 1.

### CCI model

Based on previous studies, CCI was performed to produce a severe contusion in the right sensorimotor cortex and above the hippocampus (centre of the impact: A/P, − 2.00 mm; M/L, 2.50 mm from bregma), with pronounced behavioural deficits but no mortality [34]. Following craniotomy, a CCI model was established with a TBI-0310 TBI model system (Precision Systems and Instrumentation, Fair fax, VA, USA) and the impact parameters were set as follows: 5 m/s velocity, 100 ms dwelling time, 2 mm depth and using a 3 mm diameter impactor. A pneumatic impactor was used to provide the power for impacting (Jun-Air Model 3–4). Sham mice underwent only craniotomy without CCI. Body temperature was maintained at 37.5 ± 0.5 °C with a feedback-controlled heating pad (69001, RWD Life Science, China).

### Intravenous injections

As previously described [36], at 1 h after CCI, intravenous injections of COG1410 were performed by tail vein injection of 5 μL of a 0.2 mg/mL solution of COG1410 in lactated Ringer’s solution per gram of body weight for a final dose of 1 mg/kg.

### Experimental protocols

All the experimental protocols and setups are shown in Fig. 1.

#### Experiment 1

To determine the expression time course and cellular localization of endogenous TREM2 at the injury site after CCI, thirty WT mice were randomly distributed into five groups: sham, 12 h, 24 h, 3 d, and 7 d after CCI (*n* = 6 per group). Western blot analysis was performed to evaluate the changes in TREM2 expression. Another three WT mice were assigned to the CCI 3 d group for immunofluorescence staining.

#### Experiment 2

To evaluate the effect of the TREM2 activator- COG1410 on CBF and short-term neurological function, 36 WT mice were randomly assigned to three groups: Sham, CCI + Vehicle, and CCI + COG1410 (*n* = 12 per group). In each group, we randomly sampled six mice to detect cerebral blood flow (CBF) changes by a laser speckle contrast imaging (LSCI) device. All mice were subjected to NSS scoring, wire grip tests, and rotarod tests to evaluate short-term neurological function.

#### Experiment 3

To estimate the effect of COG1410 on neurological behaviour and brain electrophysiological activity at 2 weeks after CCI, 54 WT mice were randomly assigned to three groups: Sham, CCI + Vehicle, and CCI + COG1410 (*n* = 18 per group). In each group, eight mice were randomly selected to undergo the beam walking test, the open field test, and the Morris water test. Another six mice were randomly selected to undergo motor evoked potential measurements. Local field potential monitoring was performed on the last four mice.

#### Experiment 4

To assess the effect of COG1410 on BBB disruption and brain oedema, 81 WT mice were randomly assigned to three groups: Sham, CCI + Vehicle, and CCI + COG1410 (*n* = 27 per group). In each group, six mice were used in the EB assay, three mice were used in EB fluorescence observation, six mice were assigned to Western blot analysis, six mice were assigned to immunofluorescence staining, and the last six mice were used in magnetic resonance imaging (MRI) and brain oedema analysis.

#### Experiment 5

To explore the effect of COG1410 on neuroinflammation and neural apoptosis, 36 WT mice were randomly assigned to three groups: Sham, CCI + Vehicle, and CCI + COG1410 (*n* = 12 per group). Western blot analysis was performed to detect neuroinflammation- and neural apoptosis-associated proteins using six mice per group. At the same time, immunofluorescence staining was performed to detect neuroinflammation- and neural apoptosis-associated markers using the last six mice per group.

#### Experiment 6

To determine whether the Akt/CREB/BDNF axis participated in the neuroprotective effects of TREM2 activation, 36 WT mice were randomly distributed into three groups: Sham, CCI + Vehicle, and CCI + COG1410 (*n* = 12 per group). Western blot analysis was performed to explore the effect of TREM activation on the Akt/CREB/BDNF signalling axis using six mice per group. At the same time, immunofluorescence staining was performed to detect BDNF expression using the last six mice per group. In addition, to further verify that the Akt/CREB/BDNF signalling axis participated in the protective effects of TREM2 activation and to explore whether TREM2 depletion could abolish the effects of COG1410 on vascular phenotype and microglial states, 24 WT mice were randomly distributed into two groups: WT CCI + Vehicle, and WT CCI + COG1410 (*n* = 12 per group). Twenty-four TREM2 KO mice were randomly distributed into two groups: KO CCI + Vehicle, and KO CCI + COG1410 (*n* = 12 per group). Six mice were randomly selected from each group to undergo NSS scoring, wire grip tests, and rotarod tests to evaluate short-term neurological function. After neurological assessment, Western blot analysis was performed to detect the TREM2 depletion effect on the Akt/CREB/BDNF signalling axis and BBB disruption. Immunofluorescence staining was performed on the other six mice in each group to determine whether activation of the Akt/CREB/BDNF signalling axis occurred in neurons and/or in microglia, as well as to determine vascular phenotypes and microglial states.

#### Experiment 7

To estimate the effects of TREM2 knockout on the final neurological behaviour after CCI, 12 WT mice were randomly distributed into two groups: WT CCI + Vehicle, and WT CCI + COG1410 (*n* = 6 per group), and twelve TREM2 KO mice were randomly distributed into two groups: KO CCI + Vehicle, and KO CCI + COG1410 (*n* = 6 per group). The open field test and Morris water maze were performed until 2 weeks after CCI.

### Western blot analysis

Western blot analysis was performed as previously described [34]. In brief, brain tissue including the injury site (approximately 5 mm * 5 mm * 3 mm, as shown in the red box of Fig. 4A) was collected for total protein extraction using RIPA lysate and protease and phosphatase inhibitors. The sample proteins (20 μg/lane) were separated by sodium dodecyl sulfate–polyacrylamide gel electrophoresis (SDS-PAGE) (Invitrogen) and transferred onto polyvinylidene fluoride (PVDF) membranes (Millipore, Boston, MA, USA). Membranes were blocked with 5% nonfat milk for 1 h at room temperature, then incubated overnight at 4 °C with primary antibodies, including rabbit monoclonal anti-β-actin (1:5000, Cat# ab213262, Abcam), rabbit monoclonal anti-TREM2 (1:1000, Cat# 91068S, Cell Signaling Technology), rabbit Polyclonal anti-ZO-1 (1:1000, Cat# 21773-1-AP, Proteintech), rabbit Polyclonal anti-Occludin (1:1000, Cat# 27260-1-AP, Proteintech), rabbit Polyclonal anti-Claudin-5 (1:500, Cat# 34-1600, ThermoFisher), rabbit Polyclonal anti-TNF-α (1:1000, Cat# 17590-1-AP, Proteintech), rabbit monoclonal anti-IL-1β (1:1000, Cat# ab254360, Abcam), rabbit monoclonal anti-Bcl-2 (1:1000, Cat# ab182858, Abcam), rabbit monoclonal anti-Bax (1:1000, Cat# ab182733, Abcam), rabbit Polyclonal anti-Caspase-3 (1:1000, Cat# 19677-1-AP, Proteintech), rabbit Polyclonal anti-p-Akt (1:1000, Cat# 28731-1-AP, Proteintech), rabbit Polyclonal anti-Akt (1:1000, Cat# 10176-2-AP, Proteintech), rabbit monoclonal anti-p-CREB (1:1000, Cat# ab32096, Abcam), rabbit monoclonal anti-CREB (1:1000, Cat# ab32515, Abcam), rabbit Polyclonal anti-BDNF (1:1000, Cat# 28205-1-AP, Proteintech). After being washed in Tris-buffered saline/Tween-20, the membranes were incubated for 1 h at room temperature with horseradish peroxidase-conjugated AffiniPure goat anti-rabbit IgG (1:10,000; Cat# SA00001-2, Proteintech). Enhanced chemiluminescence was used to detect proteins in the membranes (ECL Plus, Millipore), and proteins were quantified using the ImageJ software (ImageJ 1.4, NIH, Bethesda, MD, USA). All raw Western blot bands are shown in Additional file 1: Fig. S3–S9.

### Immunofluorescence staining

**Fig.2:**
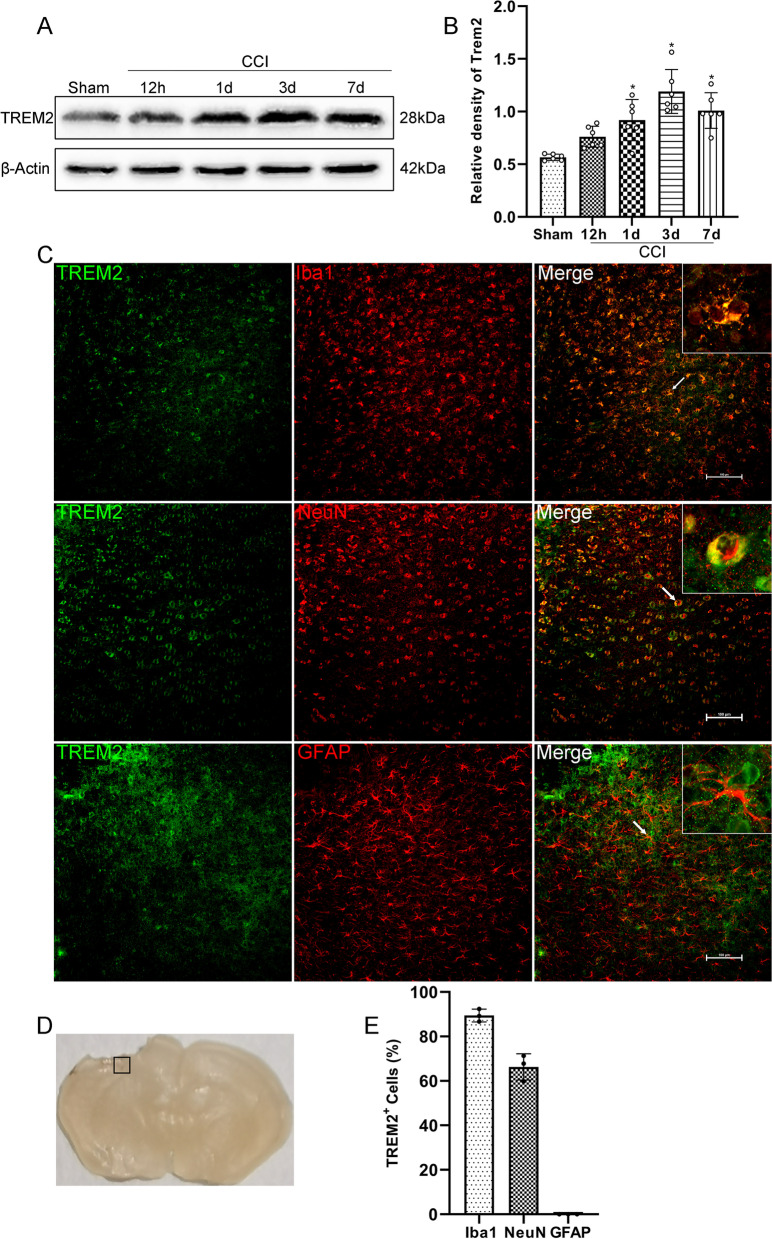
Time course and cellular localization of TREM2. **A** Representative Western blot bands of TREM2 expression at the lesion site over time. **B** Quantitative analysis of TREM2 time course expression. *p < 0.05 vs. Sham group. *n* = 6 per group. **C** Representative images of the colocalization of TREM2 (green) with microglia (Iba1, red), neurons (NeuN, red), and astrocytes (GFAP, red) at the lesion site at 3 d after CCI. Nuclei were stained with DAPI (blue). Scale bar = 100 μm, *n* = 3. **D** Brain sample with schematic illustration showing the area (indicated by black box) used for immunofluorescence analysis. **E** Quantitative analysis of the percentage of TREM2-Iba1^+^ cells to Iba1^+^ cells, TREM2-NeuN^+^ cells to NeuN^+^ cells, and TREM2-GFAP^+^ cells to GFAP^+^ cells. *n* = 3

As previously reported [37], the mice were killed under deep anaesthetization and then perfused with PBS and 4% paraformaldehyde (PFA) for fixation. The collected brains were postfixed overnight at 4 °C in 4% paraformaldehyde and then cryoprotected in graded sucrose (20% and 30%). Next, the brains were embedded in optimal cutting temperature compound and cut into 20-μm coronal sections. After washing with PBS and PBS + 0.4% Triton X-100, the brain sections were blocked with 10% goat serum for 1 h at 37 °C, incubated with primary antibodies overnight at 4 °C and washed three times with PBS. Then, the sections were incubated with secondary antibodies (1:400, Beyotime Institute of Biotechnology, Shanghai, China) conjugated to Alexa Fluor 488/594 for 1 h at room temperature. Cell nuclei were stained with 4′,6-diamidino-2-phenylindole (DAPI; Sigma-Aldrich). The primary antibodies included rabbit monoclonal anti-TREM2 (1:200, Cat# 91068S, Cell Signaling Technology), goat polyclonal anti-Iba1 (1:200, Cat# ab5076, Abcam), mouse monoclonal anti-NeuN (1:100, Cat# 66836-1-Ig, Proteintech), mouse monoclonal anti-GFAP (1:400, RRID# AB_396366, BD Biosciences), rabbit polyclonal anti-Claudin-5 (1:100, Cat# 34-1600, ThermoFisher), mouse monoclonal anti-CD31 (1:50, Cat# GTX20218, Genetex), rabbit monoclonal anti-Myeloperoxidase (MPO) (1:100, Cat# ab208670, Abcam), rabbit polyclonal anti-BDNF (1:200, Cat# 28205-1-AP, Proteintech), rabbit polyclonal anti-CD-86 (1:200, Cat# 13395-1-AP, Proteintech), rabbit polyclonal anti-CD-206 (1:200, Cat# 18704-1-AP, Proteintech), rabbit polyclonal anti-p-Akt (1:200, Cat# 28731-1-AP, Proteintech), and rabbit monoclonal anti-p-CREB (1:400, Cat# ab32096, Abcam). TUNEL assays were performed using a one-step TUNEL kit (Cat# C1090, Beyotime Institute of Biotechnology, Shanghai, China) according to the manufacturer’s instructions. For each sample, 3–4 corresponding sections at intervals of 300 μm were collected. For each group, sections from 3 to 6 mice were used for analysis. Each measurement was expressed as the average of all section measurements per mouse. All sections were collected where the lesions were located. The regions of interest (ROIs) where the images were captured are shown in the black box of Fig. 2D. All images were captured using a Leica DM4 B fluorescence microscope (Leica, DM4 B, Wetzlar, Hesse, Germany) with a 10× eyepiece and a 20× objective, and we set the same exposure time when quantitative analysis was involved in each experiment. Plugin Coloc 2 of the ImageJ software (ImageJ 1.4, NIH, Bethesda, MD, USA) was used to analyse the colocalizations of immunofluorescence. The immune-positive cell numbers were calculated with ImageJ software (ImageJ 1.4, NIH, Bethesda, MD, USA), and presented as the mean number of cells per high power field (HPF) for single staining or presented as the percentage of the number of cells for the main marker to the number of cells for the secondary marker per HPF for double-staining. The relative immunofluorescence intensity of Claudin-5 was calculated by the percentage of immunofluorescence intensity of Claudin-5 relative to immunofluorescence intensity of CD-31 with ImageJ software (ImageJ 1.4, NIH, Bethesda, MD, USA). Additionally, the relative immunofluorescence intensity of BDNF was also calculated by the percentage of immunofluorescence intensity of BDNF relative to immunofluorescence intensity of DAPI with ImageJ software (ImageJ 1.4, NIH, Bethesda, MD, USA). All counts were obtained in a blinded fashion.

### Microglia morphology analysis

Three images from Iba1-stained sections of each sample were acquired at random points surrounding the lesion sites. Endpoints of identifiable microglia were counted by plugins (AnalyzeSkeleton and FracLac) of ImageJ software (ImageJ 1.4, NIH, Bethesda, MD, USA) according to previously established protocol [38].

### Laser speckle contrast imaging

To assess the CBF changes after CCI, we used a laser speckle contrast imaging (LSCI) system (Peri-Cam PSI System; Perimed) [39]. After deep anaesthetization, the head was fixed in a stereotaxic frame (RWD Life Science Co., Ltd, Shenzhen, China), and the eyes were coated with erythromycin eye ointment. Then, the skull was exposed by a midline skin incision. Mineral oil was applied to avoid skull dryness. LSCI was performed before and after CCI at different time points (before, 6 h, 1 d, and 3 d). At each time point, the mice were continuously monitored for 5 min. We selected the injury site (a circle with 5 mm diameter) as the region of interest (ROI), and blood fluxes were measured at the ROI and expressed as perfusion units (PUs).

### Neurobehaviour assessment

The investigators were blinded to the experimental groups for all tests.

### NSS scores

As previously reported [40], the NSS scores measured general behaviour, alertness, balance and motor ability, using ten different tasks. One point was obtained for each failed task. Zero points represented the minimum deficit, and ten points represented the maximum deficit.

### Wire grip test

A 45-cm-long, 3-mm-diameter metal wire was suspended 45 cm above the ground with two vertical wooden sticks. Mice were placed in the middle of the wire to be observed for 60 s, and the latency to fall was recorded and assessed [40].

### Rotarod test

In brief, the rotating speed was started from 0 rpm; and accelerated by 3 rpm every 10 s until the rotating speed reached 30 rpm. The test ended when mice fell from the rod, and the latency was recorded and assessed [41].

### Beam walking test

In the beam walking test, the mice were placed at one end of a wooden beam (12 mm in diameter, 1 m long, and 50 cm high) and allowed to traverse the beam into a black box located at another side of the beam driven by their inner phobotaxis. The number of left foot faults and the time to transverse the whole beam was recorded. A foot slip was defined as the left paw slipping off the beam surface [37].

### Open field test

As previously reported [42], anxiety-like behaviour was evaluated 2 weeks after CCI in an open field apparatus (100 cm × 100 cm × 40 cm white box). Briefly, a mouse was placed in the centre of the apparatus, and the activity was measured and recorded for 5 min using a video-based tracking system (ANY-maze, Stoelting, USA). The total time in the centre region and the total motor distance through the whole recording process were analysed.

### Morris water maze test

Cognitive function was assessed using the Morris water maze test over 6 consecutive days. In brief, latency to find the platform were measured from days 15 to 19 after CCI using the navigation test. The time spent in the correct quadrant was measured at days 20 after CCI using the probe trial test [40]. The swim speed was also recorded to assess motor skills. The data from the spatial learning test and memory test were recorded and analysed using a video-based tracking system (ANY-maze, Stoelting, USA).

### Electrophysiological recording

#### Motor evoked potential

**Fig. 3:**
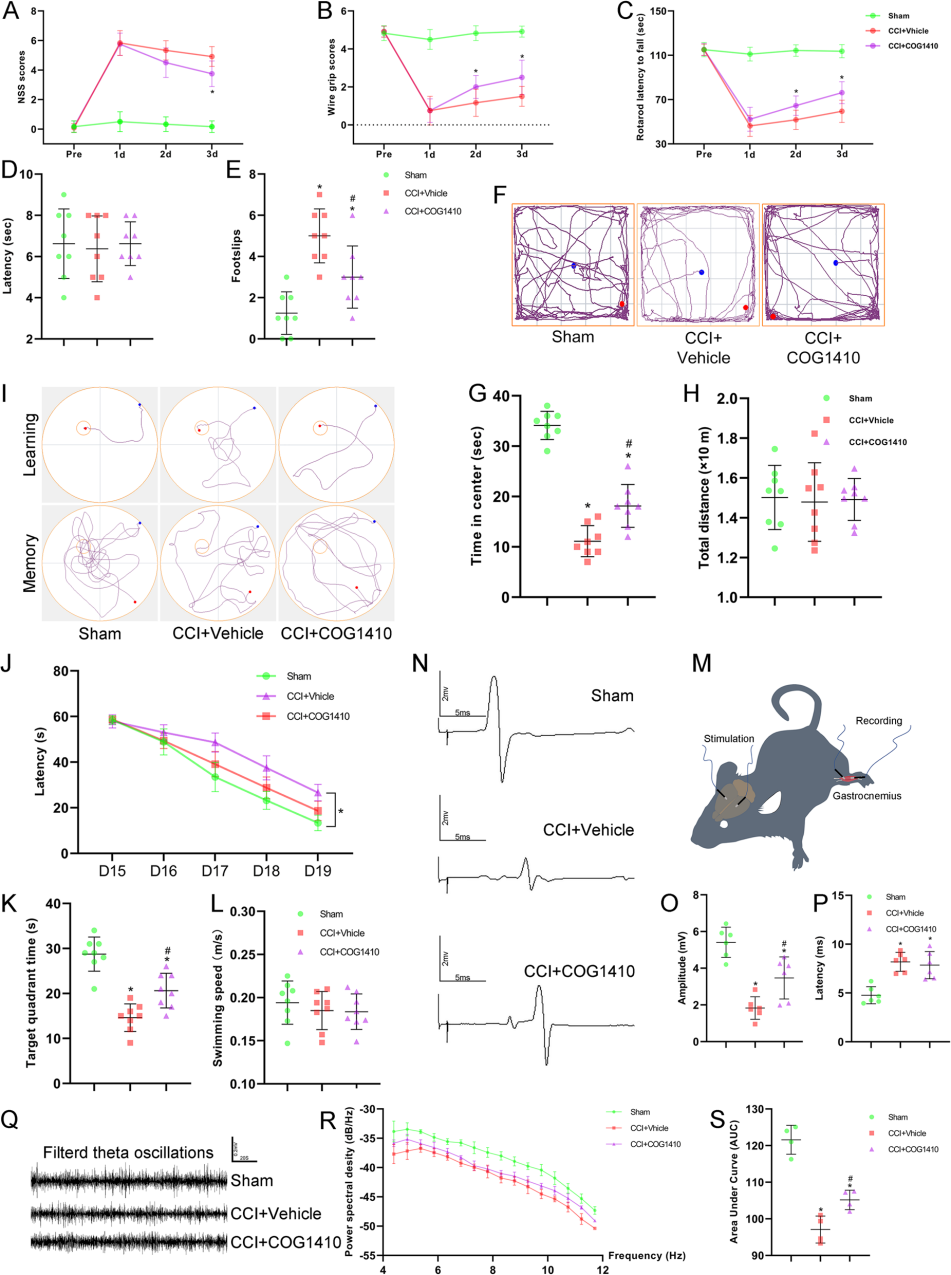
COG1410 treatment promoted neurological function and brain electrophysiological activity recovery. **A**–**C** Quantitative analysis of short-term neurological function by NSS scores (**A**), wire grip scores (**B**), and rotarod test (**C**). *p < 0.05 vs. CCI + Vehicle, *n* = 12 per group. **D**, **E** Quantitative analysis of motor function at 13 d after CCI by the beam walking test. *p < 0.05 vs. Sham; ^#^*p* < 0.05 vs. CCI + Vehicle, *n* = 8 per group. **F** Representative images of the trajectory of mice in the open field test at 14 d after CCI. **G**, **H** Quantitative analysis of centre time (**G**) and total distance travelled by mice in the open field test (**H**). *p < 0.05 vs. Sham; ^#^*p* < 0.05 vs. CCI + Vehicle, *n* = 8 per group. **I** Representative images of the trajectory of mice in the Morris water maze. **J**–**L** Quantitative analysis of latency in the learning test (**J**), target quadrant time (**K**), and average swimming speed in the target quadrant test (**L**). *p < 0.05 in **J** represents ANOWA; *p < 0.05 in **K** vs. Sham; ^#^*p* < 0.05 in **K** vs. CCI + Vehicle, *n* = 8 per group. **M** Schematic diagram of motor evoked potential (MEP) monitoring in mice. **N** Representative images of MEP waves in different groups. **O**, **P** Quantitative analysis of MEP amplitude (**O**) and MEP latency (**P**). *p < 0.05 vs. Sham; ^#^*p* < 0.05 vs. CCI + Vehicle, *n* = 6 per group. **Q** Representative images of filtered theta oscillations in different groups. **R** Representative power spectral density (PSD) curves of theta oscillations in different groups. **S.** Quantitative analysis of the total PSD of theta oscillations by the area under the curve (AUC). *p < 0.05 vs. Sham; ^#^*p* < 0.05 vs. CCI + Vehicle, *n* = 4 per group

As shown in Fig. 3M, animals were anaesthetized with pentobarbital sodium (40 mg/kg, i.p.), and two 30-G stimulating electrodes were placed in the bilateral motor cortex. The recording electrodes were placed in the left gastrocnemius muscle. Motor evoked potential (MEP) was elicited by a stimulator with a pulse of 1 ms at 7 mA (Keypoint, Medtronic, USA). The electrical stimulation was repeated at least five times in each mouse with an interval of 15 s. The base-to-peak amplitude of a single stimulation was recorded as the MEP. The MEP amplitude and latency were recorded for analysis [42, 43].

#### Local field potential

At 5 d before recording, we placed electrode tungsten wires (A-M systems, #795500) above the hippocampus on the left hemisphere of mice. In brief, the CA1 position (A/P, − 2.30 mm; M/L, 1.60 mm; D/V, − 1.50 mm from bregma) was marked according to Paxinos and Franklin’s mouse brain atlas. Four screws (diameter 1 mm, length 4 mm) were anchored on the skull. The hole for the electrode tungsten wires was then drilled, and the dura was removed carefully. Then, the electrode was inserted carefully into CA1. Finally, the electrode and screws were fixed to the skull with dental cement. The mice were allowed to recover for 5 d before recording. On the day of recording, all mice were awake and freely moving in a familiar cage. A multichannel electrophysiological system (CerePlex Direct) received the digital signal at a sampling rate of 1 kHz. LFP data were amplified and low-pass filtered at 250 Hz. We monitored for 15 consecutive min and the data of last 10 min were used to further analysis. NeuroExplorer Version 5.201 (Nex Technologies, Littleton, MA) was used to analyse all the LFP data. We used power spectral density (PSD) analysis for continuous variables to calculate the PSD of theta oscillations (4–12 Hz). The area under curve (AUC) represents the sum PSD of theta oscillations [39].

### Blood brain barrier permeability assays

To measure BBB permeability, 2% Evans Blue (EB, 4 mL/kg) was injected through the tail vein 1 h before the mice were killed. The mice were transcardially perfused with PBS, and their brains were dissected and weighed. The samples were then homogenized in PBS (1 ml/300 g), sonicated for 2 min, and centrifuged at 15,000 rpm for 5 min at 4 °C, and the supernatant was then collected in aliquots. Next, 500 μL of 50% trichloroacetic acid was added to each 500 μL of supernatant and incubated overnight at 4 °C. Finally, these samples were centrifuged at 15,000 rpm for 30 min at 4 °C. The samples were detected with a spectrometer at 610 nm and quantified using a standard curve that was normalized to tissue weight (μg/g). Then, to observe EB fluorescence, the brains were sectioned into 20 μm coronal brain sections. Red fluorescence of EB was observed as previously described [37].

### Magnetic resonance imaging

Magnetic resonance imaging (MRI) scanning was used to estimate brain oedema on a 7.0 T animal scanner (Bruker Biospin, Germany) at 3 d after CCI. The setup parameters were as follows: repetition time, 3000 ms; echo time, 30 ms; field of view, 30 × 30 mm^2^; image matrix, 256 × 256; slice thickness, 0.5 mm. Brain oedema volume was quantified by measuring the T2-hyperintense area using Weasis software [37].

### Brain water content measurement

The wet/dry method was used to measure brain water content as previously described [27]. Briefly, after deep anaesthetization and euthanasia, the brains were immediately removed and divided into three parts: the ipsilateral and contralateral hemisphere, and the cerebellum. Each part was immediately weighed to determine the wet weight and then dried at 100 °C for 24 h to obtain the dry weight. Brain water content was calculated using the following formula:

$$\left[ {{{\left( {{\text{wet weight}}\, - \,{\text{dry weight}}} \right)} \mathord{\left/ {\vphantom {{\left( {{\text{wet weight}}\, - \,{\text{dry weight}}} \right)} {\text{wet weight}}}} \right. \kern-\nulldelimiterspace} {\text{wet weight}}}} \right] \times 100\% .$$

### Statistical analysis

All results are presented as the means ± standard deviations (SDs). Before analysis, the Shapiro–Wilk test was used to test the normality of the variables. Two-way repeated-measures ANOVA with Tukey’s post hoc multiple-comparisons test was used to analyse continuously measured data. One-way analysis of variance (ANOVA) was used to compare means of different groups followed by a Tukey post hoc multiple-comparisons test. All statistical analyses were performed using GraphPad Prism software (version 9.1.0, CA, USA). Statistical significance was defined as *p* < 0.05.

## Results

### Time course expression and cellular localization of TREM2 after CCI

Western blot analysis was used to assess the endogenous TREM2 expression at 0 h (Sham), 12 h, 1 d, 3 d, and 7 d after CCI in the ipsilateral/right hemisphere. We found that in the Sham group, TREM2 was expressed at low levels, but it was activated after CCI. Compared to Sham group, TREM2 was significantly increased at 1 d, and reached to the highest level at 3 d after CCI (*p* < 0.05, Fig. 2A, B). At 7 d after CCI, the expression of TREM2 was significantly higher than that in the Sham group (*p* < 0.05, Fig. 2A, B). However, there were no significant differences between the 7 d and 3 d groups (1.01 ± 0.17 versus 1.19 ± 0.21, *p* = 0.29). Then, we selected 3 d after CCI to perform double immunofluorescence to determine the cellular localization of TREM2. The results showed that 89.43% of microglia expressed TREM2, 66.3% of neurons expressed TREM2, and no astrocytes expressed TREM2 in the peri-trauma area at 3 d after CCI (Fig. 2C, E).

### TREM2 activation promoted neurological function and brain electrophysiological activity recovery

COG1410 treatment significantly decreased the NSS scores at 3 d (*p* < 0.05, Fig. 3A), increased wire grip scores at 2 d and 3 d (*p* < 0.05, Fig. 3B), and increased rotarod falling latency at 2 d and 3 d (*p* < 0.05, Fig. 3C) after CCI when compared with the CCI + Vehicle group.

Next, we conducted a beam walking test at 13 d after CCI to assess motor function. We found that there were no significant differences between the Sham, CCI + Vehicle, and CCI + COG1410 groups in the latency with which mice crossed the whole beam (*p* > 0.05, Fig. 3D). However, CCI caused more footslips in the CCI + Vehicle group than in the Sham group (*p* < 0.05, Fig. 3E). Moreover, COG1410 treatment decreased the number of footslips compared with that in the CCI + Vehicle group (*p* < 0.05, Fig. 3E); but was still higher than that in the Sham group (*p* < 0.05, Fig. 3E).

Next, the open field test (OFT) was used to assess anxiety behaviour at 14 d after CCI. Our results showed that CCI caused less time spent in the centre in the OFT than Sham treatment, indicating that mice exhibited anxiety behaviour after CCI (*p* < 0.05, Fig. 3F, G). However, COG1410 reversed this effect in that it increased the centre time compared with the CCI + Vehicle group (*p* < 0.05, Fig. 3F, G). In addition, consistent with our previous results, there were no significant differences between the three groups in the total distance travelled in the OFT (*p* > 0.05, Fig. 3H).

Additionally, the Morris water maze was performed to evaluate spatial learning and memory on days 15 to 20 after CCI. In the learning stage, mice in both the CCI + Vehicle and CCI + COG1410 groups took more time to find the correct target than those in the Sham group (*p* < 0.05, Fig. 3I and J). However, COG1410 treatment significantly decreased the latency in the learning test compared with the CCI + Vehicle group (*p* < 0.05, Fig. 3I and J). In the memory test stage, CCI resulted in less time spent in the correct target quadrant than that in the Sham group, but COG1410 treatment increased the target quadrant time when compared to the CCI + Vehicle group (*p* < 0.05, Fig. 3I and K). Nevertheless, all mice in the different groups had the same swimming speed in the target quadrant test (*p* > 0.05, Fig. 3L).

At the same time, motor evoked potentials (MEPs) (*n* = 6 per group) and local field potentials (LFPs) (*n* = 4 per group) were measured on the remaining ten mice of each group in the Experiment 3 to detect electrophysiological activity at 2 weeks after CCI. The MEP results showed that CCI caused deficits in amplitude and latency compared to the Sham group (*p* < 0.05, Fig. 3M–P), and COG1410 treatment rescued deficits in amplitude (*p* < 0.05, Fig. 3N, O) but not in latency (*p* > 0.05, Fig. 3N, P). The LFP results showed that the waveform of the theta oscillations was different in the three groups (Fig. 3Q). PSD analysis revealed that CCI induced lower total PSD in theta oscillations compared with the Sham group (*p* < 0.05, Fig. 3R, S), while COG1410 increased the total PSD compared to the CCI + Vehicle group (*p* < 0.05, Fig. 3S).

### COG1410 treatment attenuated BBB disruption, brain oedema, and CBF decrease after CCI

**Fig. 4:**
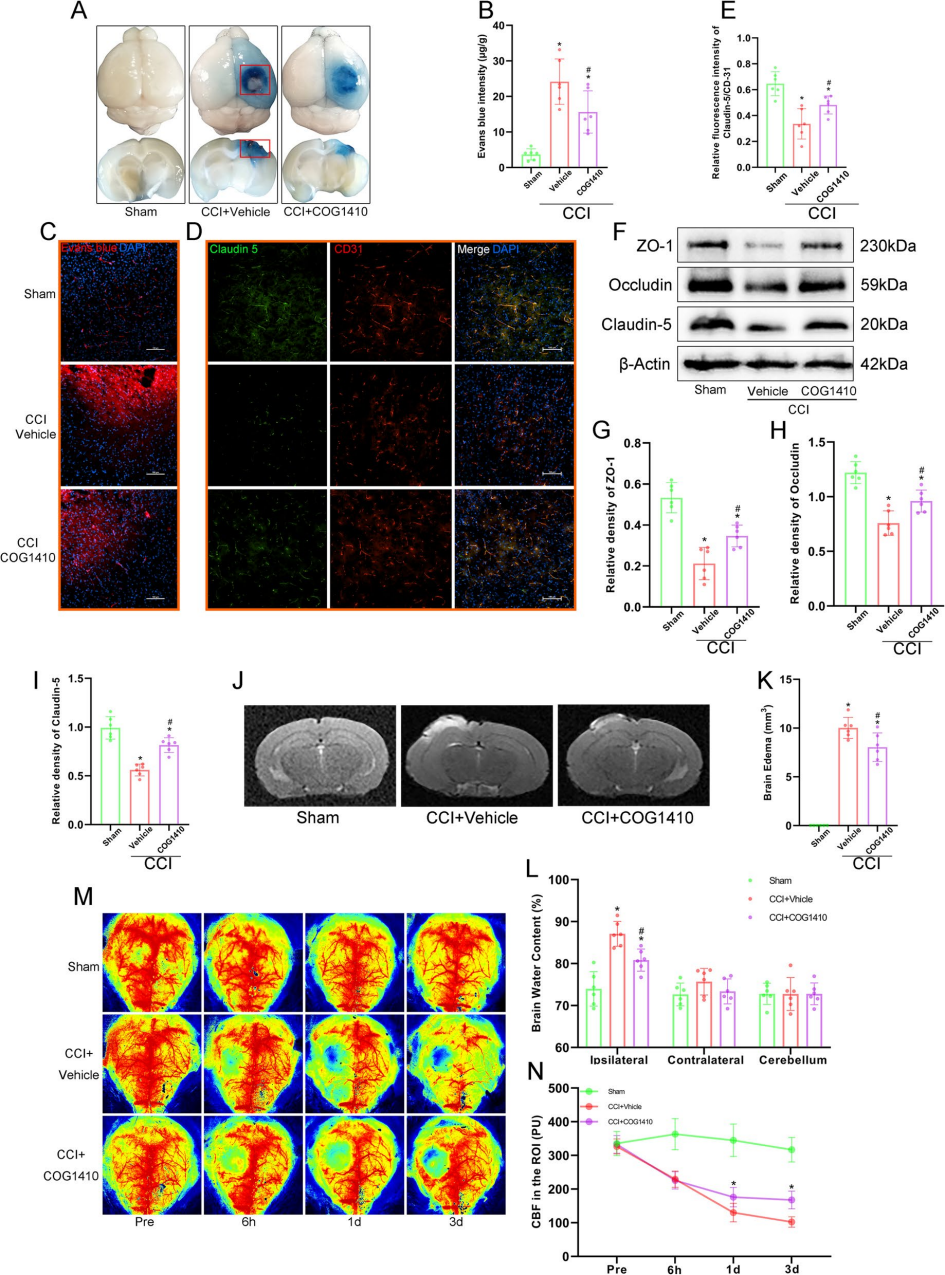
COG1410 treatment attenuated BBB disruption, brain oedema, and CBF decrease after CCI. **A** Representative horizontal and coronal images of brains after EB injection. The red boxes represent the area of brain tissue extraction in Western blot analysis. **B** Quantitative analysis of EB leakage intensity. *p < 0.05 vs. Sham; ^#^*p* < 0.05 vs. CCI + Vehicle, *n* = 6 per group. **C** Red fluorescence of EB was observed by fluorescence microscopy in different groups. Nuclei were stained with DAPI (blue). Scale bar = 100 μm. *n* = 3 per group. **D** Representative images of double immunofluorescence staining of Claudin-5 and CD31. Nuclei were stained with DAPI (blue). Scale bar = 100 μm. **E** Quantitative analysis of the relative Claudin-5 fluorescence intensity in different groups. *p < 0.05 vs. Sham; ^#^*p* < 0.05 vs. CCI + Vehicle, *n* = 6 per group. **F** Representative Western blot bands of ZO-1, Occludin, Claudin-5, and β-Actin at the lesion sites after CCI. **G**–**I** Quantitative analysis of relative ZO-1 (**G**), Occludin (**H**), and Claudin-5 (**I**) density. *p < 0.05 vs. Sham; ^#^*p* < 0.05 vs. CCI + Vehicle, *n* = 6 per group. **J** Representative images of MRI scanning at 3 d after CCI. **K** Quantitative analysis of brain oedema volume at 3 d after CCI. *p < 0.05 vs. Sham; ^#^*p* < 0.05 vs. CCI + Vehicle, *n* = 6 per group. **L** Quantitative analysis of brain water content at 3 d after CCI. *p < 0.05 vs. Sham; ^#^*p* < 0.05 vs. CCI + Vehicle, *n* = 6 per group. **M** Representative images of CBF by LSCI in different groups at different timepoints. **N** Quantitative analysis of continuous CBF changes before and after CCI. *p < 0.05 vs. CCI + Vehicle, *n* = 6 per group

In CCI mice, COG1410 visually mitigated EB dye leakage after EB injection (Fig. 4A, C). EB assays indicated that CCI caused more EB leakage than Sham treatment (*p* < 0.05, Fig. 4B). Double-immunofluorescence staining showed that Claudin-5 was decreased significantly in the CCI groups compared with the Sham group, and treatment with COG1410 attenuated this damage compared with the CCI + Vehicle group (*p* < 0.05, Fig. 4D, E). Similarly, Western blot assays revealed that the expression of BBB-associated proteins (ZO-1, Occludin, and Claudin-5) was decreased significantly after CCI compared to Sham, while COG1410 increased their expression compared to that in the CCI + Vehicle group (*p* < 0.05, Fig. 4F–I).

MRI revealed that CCI caused significant brain oedema at 3 d after CCI. And COG1410 decreased the oedema volume compared to the CCI + Vehicle group (*p* < 0.05, Fig. 4J, K). The wet/dry method showed that CCI caused severe oedema in the ipsilateral hemisphere but not in the contralateral hemisphere or cerebellum, and COG1410 alleviated ipsilateral hemisphere oedema significantly compared to the CCI + Vehicle group (*p* < 0.05, Fig. 4L).

After TBI, the CBF was significantly decreased compared with baseline [44]. Similarly, our results showed that the CBF at the traumatic injury site decreased sharply within 3 d after CCI. After COG1410 treatment, the CBF in the lesion area was significantly higher than that in the CCI + Vehicle group at 1 d and 3 d (*p* < 0.05, Fig. 4M, N), indicating that COG1410 could improve CBF after CCI in mice.

### COG1410 treatment inhibited microglial activation, neutrophil infiltration, and the expression of TNF-α and IL-1β at 3 d after CCI

**Fig. 5:**
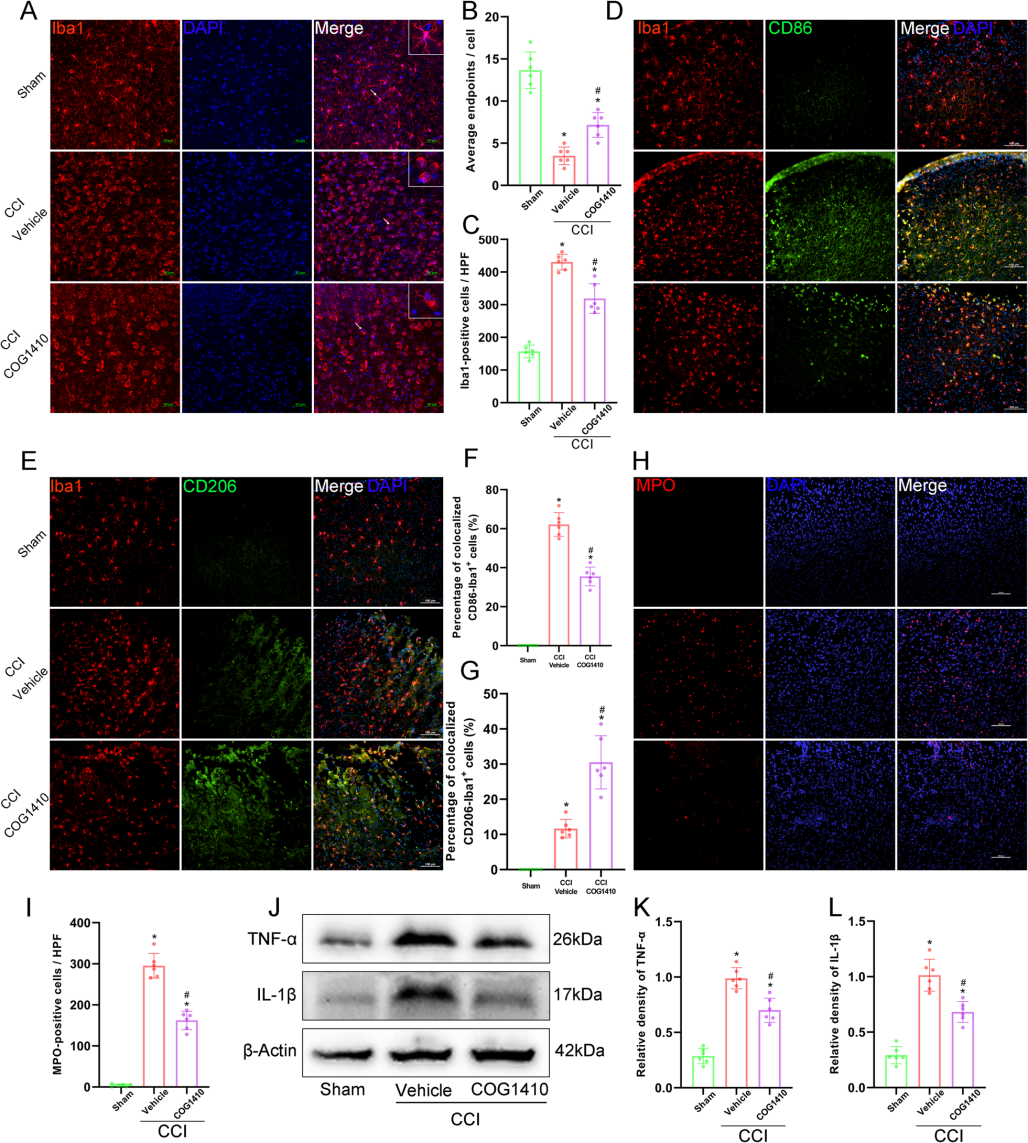
COG1410 treatment inhibited microglial activation, neutrophil infiltration, and the expression of TNF-α and IL-1β at 3 d after CCI. **A** Representative images of microglia (Iba1, red) surrounding the lesion sites. Nuclei were stained with DAPI (blue). Scale bar = 50 μm. **B**, **C** Quantitative analysis of average endpoints of Iba1-positive cells (**B**) and number of Iba1-positive cells (**C**) per HPF. HPF, high power field. *p < 0.05 vs. Sham; ^#^*p* < 0.05 vs. CCI + Vehicle, n = 6 per group. **D** Representative images of microglia (Iba1, red) and CD86-positive microglia (CD86, green) surrounding the lesion sites. Nuclei were stained with DAPI (blue). Scale bar = 100 μm. **E** Representative images of microglia (Iba1, red) and M2 microglia (CD206, green) surrounding the lesion sites. Nuclei were stained with DAPI (blue). Scale bar = 100 μm. **F**, **G** Quantitative analysis of the percentage of colocalized CD86-Iba1^+^ cells (**F**) and the percentage of colocalized CD206-Iba1^+^ cells (**G**). *p < 0.05 vs. Sham; ^#^*p* < 0.05 vs. CCI + Vehicle, *n* = 6 per group. **H** Representative images of infiltrated neutrophils (MPO, red) surrounding the lesion sites. Nuclei were stained with DAPI (blue). Scale bar = 100 μm. **I** Quantitative analysis of MPO-positive cells per HPF. *p < 0.05 vs. Sham; ^#^*p* < 0.05 vs. CCI + Vehicle, *n* = 6 per group. **J** Representative Western blot bands of TNF-α, IL-1β, and β-Actin. **K**, **L** Quantitative analysis of TNF-α (**K**) and IL-1β (**L**) density at the lesion sites after CCI. *p < 0.05 vs. Sham; ^#^*p* < 0.05 vs. CCI + Vehicle, *n* = 6 per group

Immunofluorescence staining showed that CCI caused significant microglial activation and the average endpoints of Iba1-positive cells were significantly decreased, and the numbers of Iba1-positive cells were significantly increased in the CCI groups compared with the Sham group at 3 d after CCI (*p* < 0.05, Fig. 5A–C). However, COG1410 treatment significantly increased the average endpoints of Iba1-positive cells and significantly decreased the numbers of Iba1-positive cells compared to the CCI + Vehicle group (*p* < 0.05, Fig. 5A–C). Additionally, we found that the percentage of colocalized CD86-Iba1^+^ cells was significantly increased, and the percentage of colocalized CD206-Iba1^+^ cells was decreased in the CCI groups compared to the Sham group at 3 d after CCI by immunofluorescence double-staining (*p* < 0.05, Fig. 5D–G). Nevertheless, COG1410 treatment significantly decreased the percentage of colocalized CD86-Iba1^+^ cells and increased the percentage of colocalized CD206-Iba1^+^ cells compared to the CCI + Vehicle group (*p* < 0.05, Fig. 5D–G).

At the same time, immunofluorescence staining showed that CCI caused significant neutrophil infiltration, and the numbers of MPO-positive cells were significantly increased in the CCI groups compared with the Sham group at 3 d after CCI (*p* < 0.05, Fig. 5H, I). However, COG1410 treatment significantly decreased the number of MPO-positive cells compared to the CCI + Vehicle group (*p* < 0.05, Fig. 5H, I).

In addition, the expression of the proinflammatory mediators TNF-α and IL-1β in the ipsilateral hemisphere was significantly decreased by COG1410 treatment compared with that in the CCI + Vehicle group at 3 d after CCI, as shown by Western blot assays (*p* < 0.05, Fig. 5J–L).

### COG1410 treatment attenuated neuronal apoptotic death and the expression of apoptosis related proteins at 3 d after CCI

**Fig. 6:**
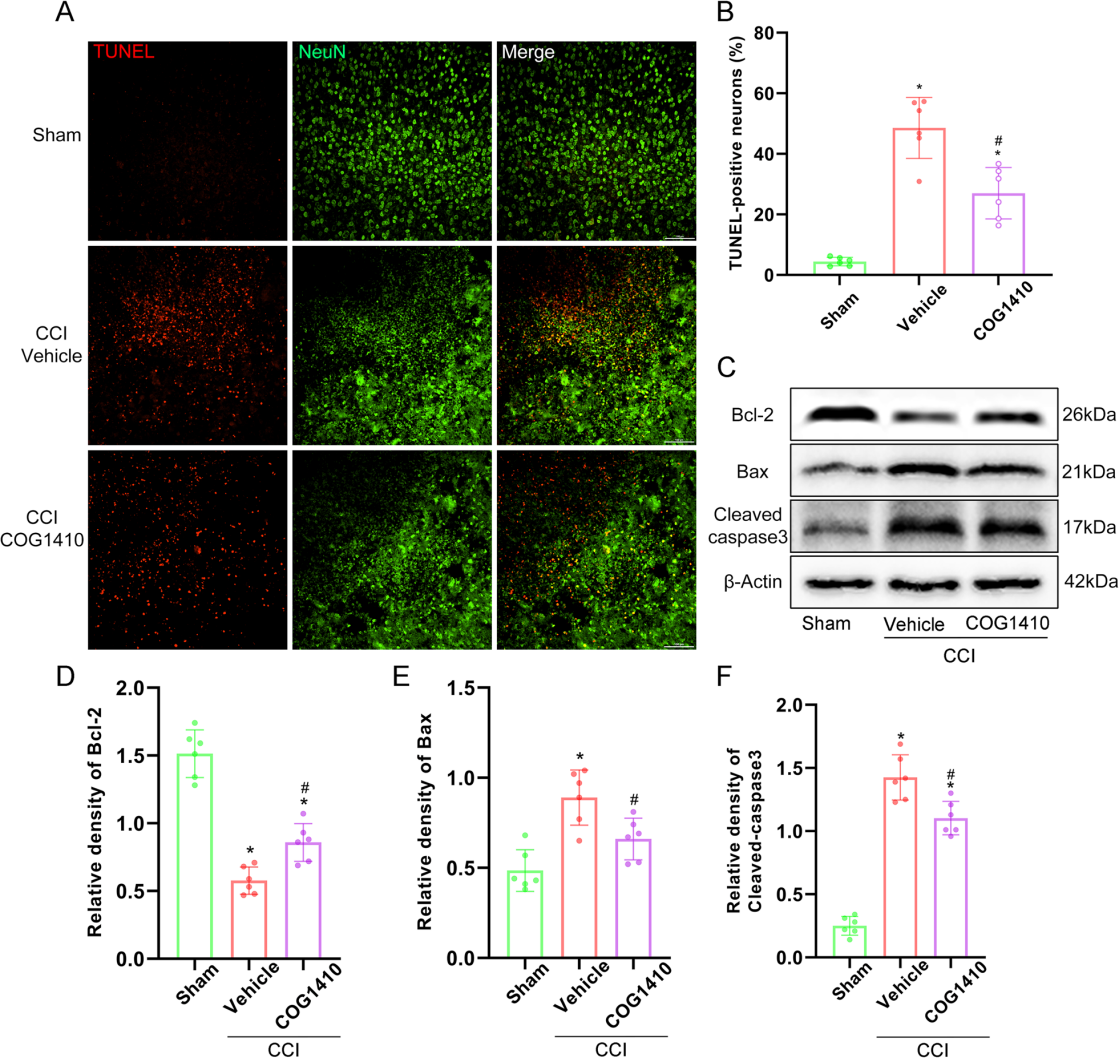
COG1410 treatment attenuated neuronal apoptotic death and the expression of apoptosis related proteins at 3 d after CCI. **A** Representative images of dead cells (TUNEL, red) and neurons (NeuN, green) surrounding the lesion sites. Scale bar = 100 μm. **B** Quantitative analysis of TUNEL-positive neurons surrounding the lesion sites at 3 d after CCI. *p < 0.05 vs. Sham; ^#^*p* < 0.05 vs. CCI + Vehicle, *n* = 6 per group. **C** Representative western blot bands of Bcl-2, Bax, cleaved-caspase-3, and β-Actin at the lesion sites after CCI. **D**–**F** Quantitative analysis of Bcl-2 (**D**), Bax (**E**), and cleaved-caspase-3 (**F**) density at the lesion sites after CCI. *p < 0.05 vs. Sham; ^#^*p* < 0.05 vs. CCI + Vehicle, *n* = 6 per group

Double-immunofluorescence staining of TUNEL and NeuN showed that there were significantly increased TUNEL-positive neurons surrounding the trauma site at 3 d after CCI, and COG1410 reduced the TUNEL-positive neurons compared to the CCI + Vehicle group (*p* < 0.05, Fig. 6A, B). Additionally, the expression of the apoptotic molecular markers Bcl-2, Bax, and cleaved-caspase-3 at 3 d after CCI was measured by Western blotting. The results showed that the expression of Bcl-2 was significantly increased and the expression of Bax and cleaved-caspase-3 was significantly decreased with COG1410 treatment when compared with the CCI + Vehicle group at 3 d after CCI (*p* < 0.05, Fig. 6C–F).

### The Akt/CREB/BDNF axis participated in the neuroprotective effects of COG1410-induced TREM2 activation

**Fig. 7:**
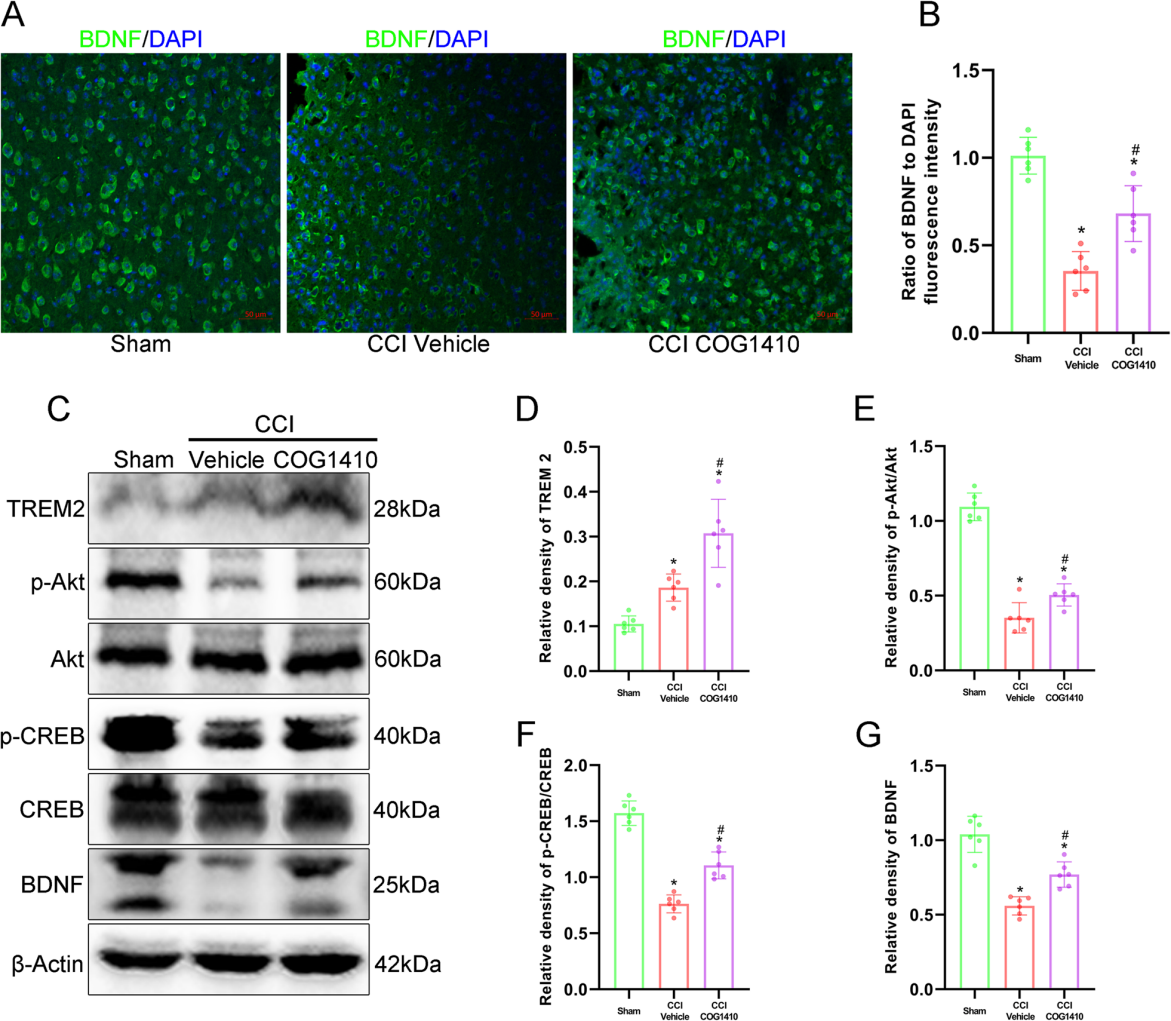
The Akt/CREB/BDNF axis participated in the neuroprotective effects of TREM2 activation. **A** Representative images of BDNF (green) surrounding the lesion sites at 3 d after CCI. Scale bar = 50 μm. **B** Quantitative analysis of the relative BDNF fluorescence intensity surrounding the lesion sites after CCI. *p < 0.05 vs. Sham; ^#^*p* < 0.05 vs. CCI + Vehicle, *n* = 6 per group. **C** Representative Western blot bands of TREM2, p-Akt, Akt, p-CREB, CREB, BDNF, and β-Actin at the lesion sites after CCI. **D**–**G** Quantitative analysis of TREM2 (**D**), p-Akt (**E**), p-CREB (**F**), and BDNF (**G**) density at the lesion sites after CCI. *p < 0.05 vs. Sham; ^#^*p* < 0.05 vs. CCI + Vehicle, *n* = 6 per group

Immunofluorescence staining showed that the neuroprotective factor BDNF was decreased at 3 d after CCI, but it was significantly increased with COG1410 treatment when compared to the CCI + Vehicle group (*p* < 0.05, Fig. 7A, B). Western blot analysis showed that after COG1410 treatment, the expression of TREM2 significantly increased when compared with both the Sham and CCI + Vehicle groups at 3 d after CCI (*p* < 0.05, Fig. 7C, D). Moreover, the expression of p-Akt, p-CREB and BDNF was significantly decreased in the CCI + Vehicle group compared with the Sham group at 3 d after CCI (*p* < 0.05, Fig. 7C, E–G). However, COG1410 treatment significantly increased the expression of p-Akt, p-CREB and BDNF when compared with the CCI + Vehicle group at 3 d after CCI (*p* < 0.05, Fig. 7C, E–G).

### TREM2 depletion abolished the effects of COG1410 on short-term neurobehaviour and the Akt/CREB/BDNF signalling pathway

**Fig. 8:**
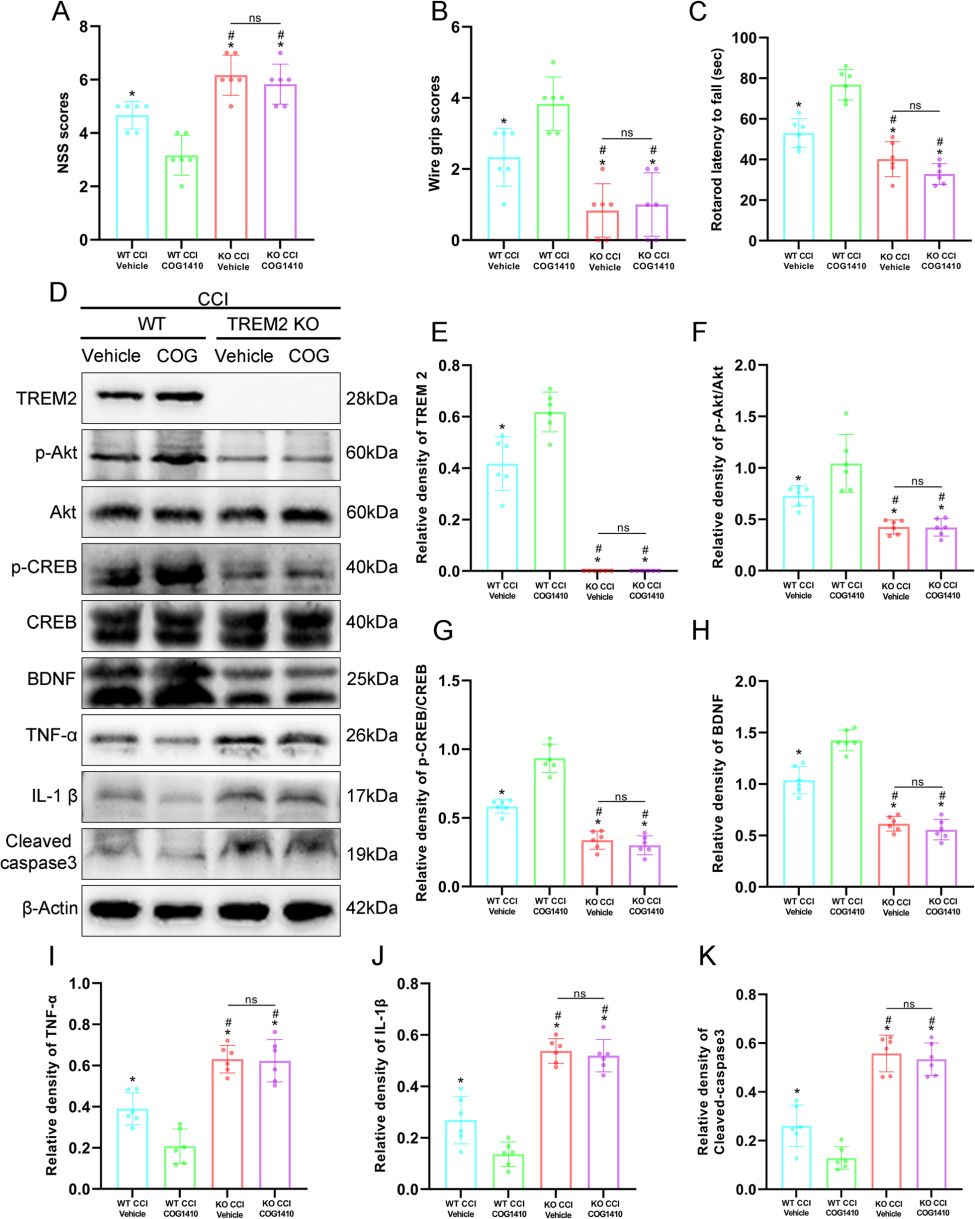
TREM2 depletion abolished the effects of COG1410 on short-term neurobehaviour and the Akt/CREB/BDNF signalling pathway. A–**C** Quantitative analysis of short-term neurological function by NSS scores (**A**), wire grip scores (**B**), and rotarod test (**C**) at 3 d after CCI. *p < 0.05 vs. WT CCI + COG1410; ^#^*p* < 0.05 vs. WT CCI + Vehicle; ns *p* > 0.05 KO CCI + Vehicle vs. KO CCI + COG1410, *n* = 6 per group. **D** Representative Western blot bands of TREM2, p-Akt, Akt, p-CREB, CREB, BDNF, TNF-α, IL-1β, cleaved-caspase-3, and β-Actin at the lesion sites after CCI. **E**–**K** Quantitative analysis of TREM2 (**E**), p-Akt (**F**), p-CREB (**G**), BDNF (**H**), TNF-α (**I**), IL-1β (**J**), and cleaved-caspase-3 (**K**) density at the lesion sites after CCI. *p < 0.05 vs. WT CCI + COG1410; ^#^*p* < 0.05 vs. WT CCI + Vehicle; ns *p* > 0.05 KO CCI + Vehicle vs. KO CCI + COG1410, *n* = 6 per group

We used TREM2 KO mice to further verify that TREM2 had neuroprotective effects and that the p-Akt/CREB/BDNF axis participated in the neuroprotective effects of TREM2 after CCI. Short-term neurological function assessments showed that NSS scores was significantly increased and the wire grip scores and rotarod falling latency were significantly decreased in the KO CCI + Vehicle and KO CCI + COG1410 groups compared with the WT CCI + Vehicle group at 3 d after CCI (*p* < 0.05, Fig. 8A–C). Intriguingly, there were no changes in NSS scores, wire grip scores, or rotarod falling latency with COG1410 treatment in the KO CCI + COG1410 group compared with the KO CCI + Vehicle group at 3 d after CCI (*p* > 0.05, Fig. 8A–C). However, the results again revealed that COG1410 treatment significantly decreased NSS scores and significantly increased wire grip scores and rotarod falling latency in the WT CCI + COG1410 group when compared to the WT CCI + Vehicle group at 3 d after CCI (*p* < 0.05, Fig. 8A–C). Western blot analysis showed that TREM2 expression was significantly increased in the WT CCI + COG1410 group compared with the WT CCI + Vehicle group, however, no TREM2 was expressed in the TREM2 KO groups at 3 d after CCI (*p* < 0.05, Fig. 8D, E). Moreover, the expression of p-Akt, p-CREB, and BDNF was significantly decreased and the expression of TNF-α, IL-1β, and cleaved-caspase-3 was significantly increased in the KO CCI + Vehicle and KO CCI + COG1410 groups compared with the WT CCI + Vehicle group at 3 d after CCI (*p* < 0.05, Fig. 8D, F–K). However, there were no significant changes in p-Akt, p-CREB, BDNF, TNF-α, IL-1β, and cleaved-caspase-3 expression in the KO CCI + COG1410 group compared with the KO CCI + Vehicle group at 3 d after CCI (*p* > 0.05, Fig. 8D, F–K). In addition, our Western blot results once again proved that COG1410 treatment significantly increased the expression of p-Akt, p-CREB and BDNF and significantly decreased TNF-α, IL-1β, and cleaved-caspase-3 expression in the WT CCI + COG1410 group compared with the WT CCI + Vehicle group at 3 d after CCI (*p* < 0.05, Fig. 8D, F–K).

### Activation of the Akt/CREB/BDNF signalling axis occurred in microglia

**Fig. 9:**
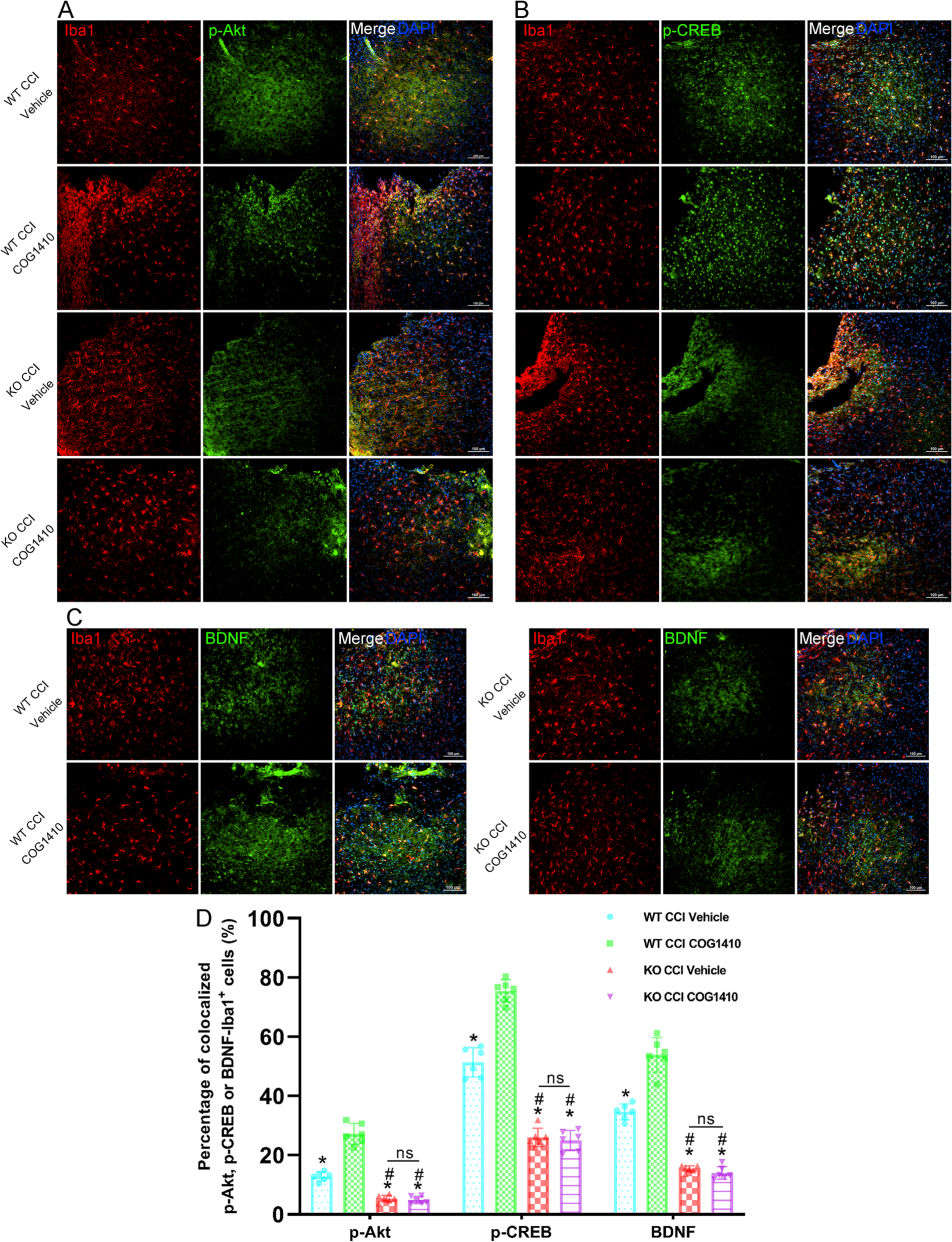
Activation of the Akt/CREB/BDNF signalling axis occurred in microglia. **A** Representative images of microglia (Iba1, red) and p-Akt^+^ cells (p-Akt, green) surrounding the lesion sites at 3 d after CCI. Nuclei were stained with DAPI (blue). Scale bar = 100 μm. **B** Representative images of microglia (Iba1, red) and p-CREB^+^ cells (p-CREB, green) surrounding the lesion sites at 3 d after CCI. Nuclei were stained with DAPI (blue). Scale bar = 100 μm. **C** Representative images of microglia (Iba1, red) and BDNF^+^ cells (BDNF, green) surrounding the lesion sites at 3 d after CCI. Nuclei were stained with DAPI (blue). Scale bar = 100 μm. **D** Quantitative analysis of the percentage of colocalized p-Akt-Iba1 + cells, p-CREB-Iba1 + cells, and BDNF-Iba1 + cells. *p < 0.05 vs. WT CCI + COG1410; ^#^*p* < 0.05 vs. WT CCI + Vehicle; ns *p* > 0.05 KO CCI + Vehicle vs. KO CCI + COG1410, *n* = 6 per group

In microglia, double immunofluorescence staining results showed that TREM2 KO significantly decreased the percentage of colocalized p-Akt-Iba1^+^ cells (*p* < 0.05, Fig. 9A, D), p-CREB-Iba1^+^ cells (*p* < 0.05, Fig. 9B, D), and BDNF-Iba1^+^ cells (*p* < 0.05, Fig. 9C, D) when compared to the WT CCI + Vehicle group at 3 d after CCI. However, there were no changes in the percentage of colocalized p-Akt-Iba1^+^ cells (*p* > 0.05, Fig. 9A, D), p-CREB-Iba1^+^ cells (*p* > 0.05, Fig. 9B, D), or BDNF-Iba1^+^ cells (*p* > 0.05, Fig. 9C, D) treated with COG1410 in the KO CCI + COG1410 group compared with the KO CCI + Vehicle group at 3 d after CCI. Intriguingly, our results showed that COG1410 treatment significantly increased the percentage of colocalized p-Akt-Iba1^+^ cells (*p* < 0.05, Fig. 9A, D), p-CREB-Iba1^+^ cells (*p* < 0.05, Fig. 9B, D), and BDNF-Iba1^+^ cells (*p* < 0.05, Fig. 9C, D) in the WT CCI + COG1410 group compared with the WT CCI + Vehicle group.

At the same time, in neurons, there were no significant differences in the percentage of colocalized p-Akt-NeuN^+^ cells (*p* > 0.05, Additional file 1: Fig. S2A, D) and p-CREB-NeuN^+^ cells (*p* > 0.05, Additional file 1: Fig. S2B, D) among all four groups. However, TREM2 KO significantly decreased the percentage of colocalized BDNF-NeuN^+^ cells (*p* < 0.05, Additional file 1: Fig. S2C, D) compared to the WT CCI + Vehicle group at 3 d after CCI and no differences were found in the percentage of colocalized BDNF-NeuN^+^ cells (*p* > 0.05, Additional file 1: Fig. S2C, D) between the KO CCI + Vehicle and KO CCI + COG1410 groups in neurons. In addition, COG1410 treatment significantly increased the percentage of colocalized BDNF-NeuN^+^ cells in the WT CCI + COG1410 group compared with the WT CCI + Vehicle group (*p* < 0.05, Additional file 1: Fig. S2C, D).

### TREM2 depletion abolished the effects of COG1410 on vascular phenotypes and microglial states and finally abolished the protective effects on neurological function

**Fig. 10:**
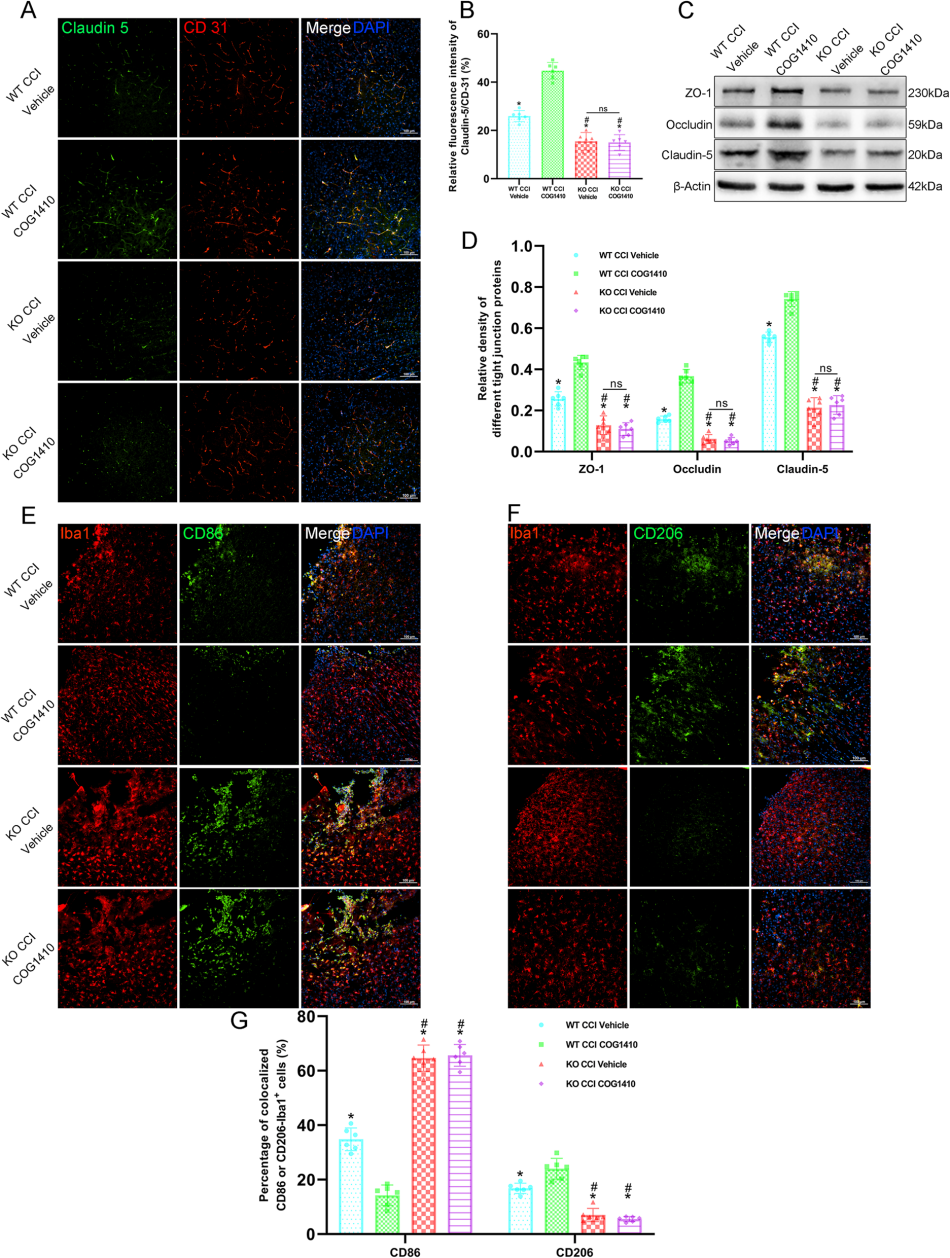
TREM2 depletion abolished the effects of COG1410 on vascular phenotypes and microglial states. **A** Representative images of Claudin-5 (green) and CD31 (red) surrounding the lesion sites at 3 d after CCI. Nuclei were stained with DAPI (blue). Scale bar = 100 μm. **B** Quantitative analysis of the relative Claudin-5 to CD31 fluorescence intensity in different groups. *p < 0.05 vs. WT CCI + COG1410; ^#^*p* < 0.05 vs. WT CCI + Vehicle; ns *p* > 0.05 KO CCI + Vehicle vs. KO CCI + COG1410, *n* = 6 per group. **C** Representative Western blot bands of ZO-1, Occludin, Claudin-5, and β-Actin at the lesion sites after CCI. **D** Quantitative analysis of relative ZO-1, Occludin, and Claudin-5 density. *p < 0.05 vs. WT CCI + COG1410; ^#^*p* < 0.05 vs. WT CCI + Vehicle; ns *p* > 0.05 KO CCI + Vehicle vs. KO CCI + COG1410, *n* = 6 per group. **E** Representative images of microglia (Iba1, red) and CD86-positive microglia (CD86, green) surrounding the lesion sites at 3 d after CCI. Nuclei were stained with DAPI (blue). Scale bar = 100 μm. **F** Representative images of microglia (Iba1, red) and CD206-positive microglia (CD206, green) surrounding the lesion sites at 3 d after CCI. Nuclei were stained with DAPI (blue). Scale bar = 100 μm. **G** Quantitative analysis of the percentage of colocalized CD86-Iba1^+^ cells and the percentage of colocalized CD206-Iba1^+^ cells in different groups at 3 d after CCI. *p < 0.05 vs. WT CCI + COG1410; ^#^*p* < 0.05 vs. WT CCI + Vehicle; ns *p* > 0.05 KO CCI + Vehicle vs. KO CCI + COG1410, *n* = 6 per group

Regarding the vascular phenotypes, double immunofluorescence staining showed that the relative fluorescence intensity of Claudin-5 to CD31 was significantly decreased in the KO CCI + Vehicle and KO CCI + COG1410 groups compared with the WT CCI + Vehicle group at 3 d after CCI (*p* < 0.05, Fig. 10A, B). However, there was no significant change in the relative fluorescence intensity of Claudin-5 to CD31 in the KO CCI + COG1410 group compared with the KO CCI + Vehicle group at 3 d after CCI (*p* > 0.05, Fig. 10A, B). Additionally, the results once again verified that COG1410 treatment significantly increased the relative fluorescence intensity of Claudin-5 to CD31 in the WT CCI + COG1410 group compared with the WT CCI + Vehicle group (*p* < 0.05, Fig. 10A, B). In addition, Western blot analysis showed that the expression of ZO-1, Occludin, and Claudin-5 was significantly decreased in the KO CCI + Vehicle and KO CCI + COG1410 groups compared with the WT CCI + Vehicle group at 3 d after CCI (*p* < 0.05, Fig. 10C, D). However, there were no significant changes in ZO-1, Occludin, and Claudin-5 expression in the KO CCI + COG1410 group compared with the KO CCI + Vehicle group at 3 d after CCI (*p* > 0.05, Fig. 10C, D). In addition, we once again proved that COG1410 treatment significantly increased the expression of ZO-1, Occludin, and Claudin-5 in the WT CCI + COG1410 group compared with the WT CCI + Vehicle group at 3 d after CCI (*p* < 0.05, Fig. 10C, D).

Regarding the microglial states, we found that the percentage of colocalized CD86-Iba1^+^ cells was significantly increased, and the percentage of colocalized CD206-Iba1^+^ cells was decreased in the KO CCI + Vehicle and KO CCI + COG1410 groups compared with the WT CCI + Vehicle group at 3 d after CCI by immunofluorescence double-staining (*p* < 0.05, Fig. 10E–G). However, there were no significant changes in the percentage of colocalized CD86-Iba1^+^ cells and CD206-Iba1^+^ cells in the KO CCI + COG1410 group compared with the KO CCI + Vehicle group at 3 d after CCI (*p* > 0.05, Fig. 10E–G). Nevertheless, COG1410 treatment significantly decreased the percentage of colocalized CD86-Iba1^+^ cells and increased the percentage of colocalized CD206-Iba1^+^ cells in the WT CCI + COG1410 group as compared to the WT CCI + Vehicle group (*p* < 0.05, Fig. 10E–G).

**Fig. 11:**
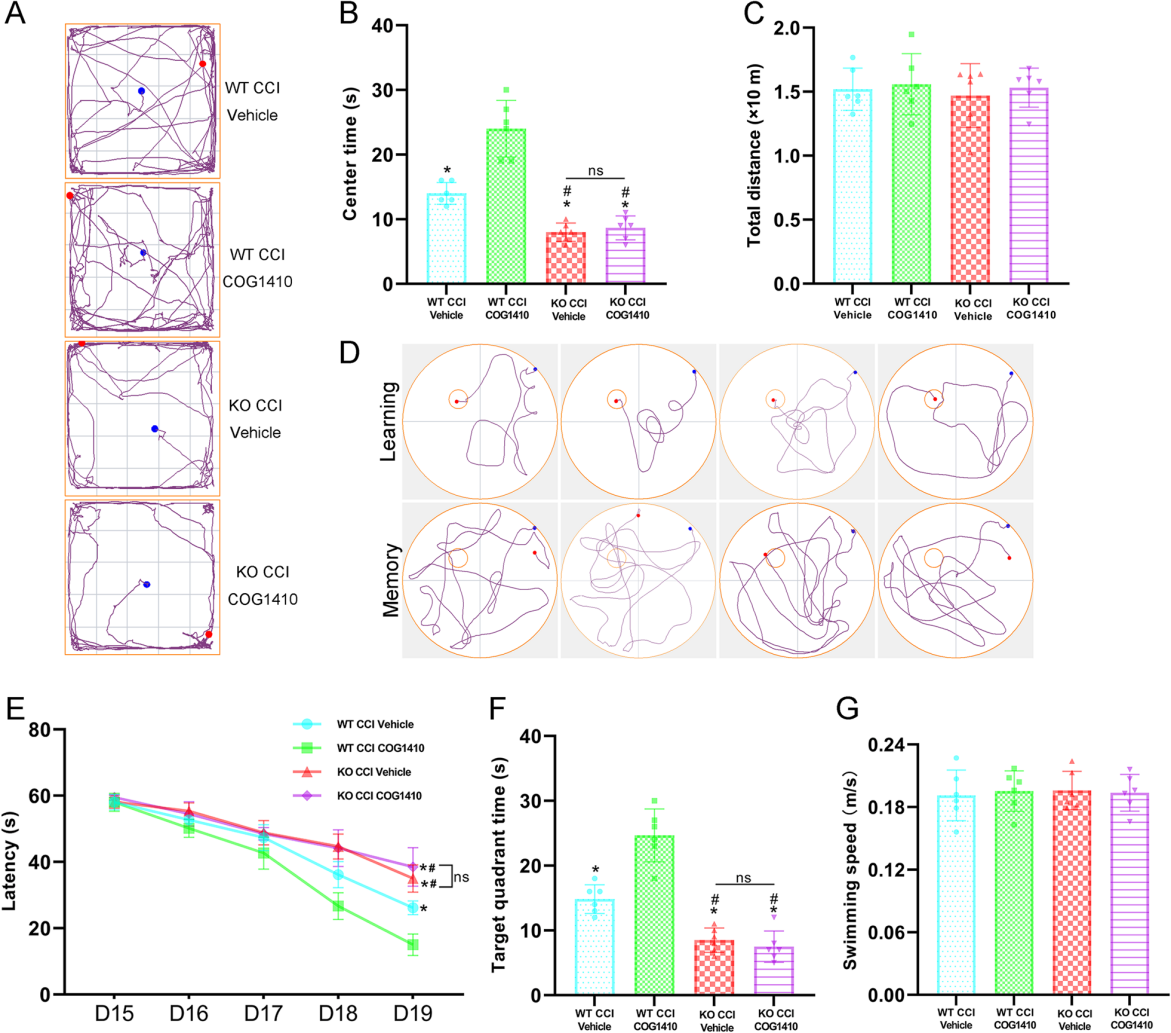
TREM2 depletion finally abolished the protective effects of COG1410 on neurological functions. **A** Representative images of the trajectory of mice in different groups in the open field test at 14 d after CCI. **B**, **C** Quantitative analysis of centre time (**B**) and total distance travelled by the mice in the open field test (**C**). *p < 0.05 vs. WT CCI + COG1410; ^#^*p* < 0.05 vs. WT CCI + Vehicle; ns *p* > 0.05 KO CCI + Vehicle vs. KO CCI + COG1410, *n* = 6 per group. **D** Representative images of the trajectory of mice in different groups in the Morris water maze. **E**–**G** Quantitative analysis of latency in the learning test (**E**), target quadrant time in the memory test (**F**), and average swimming speed in the memory test (**G**). *p < 0.05 vs. WT CCI + COG1410; ^#^*p* < 0.05 vs. WT CCI + Vehicle; ns *p* > 0.05 KO CCI + Vehicle vs. KO CCI + COG1410, *n* = 6 per group

Finally, we conducted an open field test and Morris water maze to estimate the effects of TREM2 KO on behavioural functions at 2 weeks after CCI. In the open field test, the results showed that centre time was significantly decreased in the KO CCI + Vehicle and KO CCI + COG1410 groups compared with the WT CCI + Vehicle group (*p* < 0.05, Fig. 11A, B). There were no differences between the KO CCI + Vehicle and KO CCI + COG1410 groups (*p* > 0.05, Fig. 11A, B). Once again, COG1410 treatment significantly increased the centre time in the WT CCI + COG1410 group compared with the WT CCI + Vehicle group (*p* < 0.05, Fig. 11A, B). In addition, there were no differences in the total motor distance among these four groups (*p* > 0.05, Fig. 11C). In the Morris water maze, TREM2 KO significantly increased the latency to find the correct target at 19 d in the learning stage (*p* < 0.05, Fig. 11D, E) and decreased the time in the correct target quadrant at 20 d in the memory stage (*p* < 0.05, Fig. 11D, F) when compared to the WT CCI + Vehicle group after CCI. However, there were no differences in the learning or memory stages between the KO CCI + Vehicle and KO CCI + COG1410 groups (*p* > 0.05, Fig. 11D–F). Additionally, TREM2 treatment significantly decreased the latency to find the correct target at 19 d in the learning stage (*p* < 0.05, Fig. 11D, E) and increased the time in the correct target quadrant at 20 d in the memory stage (*p* < 0.05, Fig. 11D, F) when compared to the WT CCI + Vehicle group after CCI. There were no differences in the swimming speed at 20 d among all four groups (*p* > 0.05, Fig. 11G).

## Discussion

In the present study, we were the first to investigate the neuroprotective effects of TREM2 activation by tail vein injection of an apoE mimic peptide (COG1410) and explored its possible mechanism in a model of TBI in both WT mice and TREM2 KO mice. We discovered that TREM2 activation by COG1410 improved neurological functions within 3 d, as well as neurological functions and brain electrophysiological activity at 2 weeks after CCI. It also alleviated BBB disruption, reduced brain oedema, alleviated CBF reduction, inhibited microglial activation and neutrophil infiltration, and suppressed neuronal apoptosis at the traumatic sites after CCI. In addition, we also demonstrated that the Akt/CREB/BDNF signalling pathway might participate in the neuroprotective effects of TREM2, and that COG1410 treatment could regulate the protein expression of this axis. Moreover, TREM2 elimination aggravated short-term neurological performance after CCI. In addition, TREM2 depletion further reduced p-Akt, p-CREB, and BDNF expression and further increased the expression of TNF-α, IL-1β, and cleaved-caspase-3 at 3 d after CCI. However, the usage of COG1410 did not reverse the effects caused by TREM2 KO. In addition, we found that activation of the Akt/CREB/BDNF signalling pathway in microglia participated in the neuroprotective effects of TREM2. TREM2 knockout abolished the effects of COG1410 on vascular phenotypes and microglial states, and the neuroprotective effects of COG1410 were suppressed by TREM2 depletion. Finally, our findings suggested that TREM2 activation may alleviate neural damage after CCI, which was, at least in part, mediated by activation of the Akt/CREB/BDNF signalling axis in microglia.

Secondary injury is a complicated and pivotal pathological process after TBI, and the neuroinflammatory response is one of the most important reactions involved. After TBI onset, resident microglia and macrophages are activated and elicit neuroinflammatory responses, as well as neutrophil infiltration. Resident microglia and macrophages immediately respond to trauma by sensing DAMPs, and then, initiate cytokine and chemokine release, which changes the environment of the CNS and provides a cue for neutrophil infiltration [9]. A large number of studies indicate that neuroinflammation after TBI is deleterious, however, many anti-inflammatory drug clinical trials have failed to achieve a therapeutic benefit [45]. Indeed, the CRASH trial has proven that nonspecific and high-dose inflammation suppression is harmful [46]. Not all neuroinflammation is detrimental, but at least some neuroinflammation may serve as a promoter of neural recovery [47]. In the current study, TREM2 activation partially suppressed neuroinflammation, i.e. alleviated the partial release of some proinflammatory cytokines (TNF-α, and IL-1β), indicating that the inhibition of neuroinflammation by TREM2 is a promising therapeutic target for TBI treatment. In fact, TREM2 has been proven to have protective effects in neurodegenerative diseases, fatty liver, obesity, atherosclerosis, and tumour [19]. Crossing mice deficient in TREM2 with models of neurodegeneration elucidated that TREM2 can promote microglial survival [32]. Moreover, TREM2 can promote the states of microglia to disease associated microglia (DAMs), which can restrict plaque growth in an Alzheimer’s disease model [48]. Research has demonstrated that TREM2 limits tissue destruction and facilitates repair within the CNS by clearing cellular debris during experimental autoimmune encephalomyelitis (EAE), an animal model of multiple sclerosis [22]. In the stroke research field, TREM2 has been demonstrated to have neuroprotective effects in intracerebral haemorrhage, ischaemic stroke, and subarachnoid haemorrhage [27, 28, 49–51]. TREM2 expression peaks at different time points in different stroke subtypes. In intracerebral haemorrhage, it peaks at 24 h [27]. In ischaemic stroke, it peaks at 3–7 d [49, 50]. In subarachnoid haemorrhage, it peaks at 48 h [29]. In our study, the expression of endogenous TREM2 peaked at 3 d after CCI in mice. As we mentioned before, resident microglia and macrophages are activated immediately by traumatic injury and elicit neuroinflammatory responses, as well as neutrophil infiltration [9]. Our results indicated that in the hyperacute phase of the natural course of TBI, the body itself may need some inflammation to trigger the next repair process. Consequently, only partial neuroinflammation was suppressed by COG1410-induced TREM2 activation, which might be the reason why COG1410 treatment could improve neurological function recovery after CCI in mice. In addition to restricting inflammation, TREM2 also plays a key role in different diseases by promoting phagocytosis and cell survival [19]. In brief, the effects of TREM2 are multifaceted and it is necessary to explore its exact mechanism after TBI to further guide translational research on TBI.

In a previous study by Chen et al., TREM2 activation by COG1410 alleviated neuroinflammation and neural apoptosis and ultimately improved neurological function through the PI3K/Akt signalling pathway after experimental ICH [27]. They found that after ICH, COG1410 treatment alleviated short-term brain oedema and neurological behaviour, which was consistent with our results in an experimental TBI model. After TBI, direct trauma-induced tissue loss and cell death lead to primary damage. Subsequently, neuroinflammation, BBB disruption, and other factors contribute to secondary brain injury [52]. Exorbitant neuroinflammation damages BBB integrity and BBB disruption can further amplify neuroinflammation. In addition, BBB disruption induces CBF alteration and brain oedema [53]. A previous study reported that bone marrow stromal cell-enhanced BBB reconstitution plays an important role in the recovery of CBF after TBI in rats [54]. Conversely, all these factors further magnify neuroinflammation [14–16]. In our study, we also estimated BBB integrity and CBF changes after CCI and found that COG1410 alleviated BBB disruption and that TREM2 knockout abolished these protective effects. Moreover, we also found that COG1410 improved CBF at traumatic sites at 2 d and 3 d after CCI. Next, in addition to the increased number of microglia, we also found that both the morphology and states of microglia were regulated by TREM2. Microglia have a surveillance function characterized by the continuous monitoring of central nervous system microenvironment in a ramified morphology [55]. The morphological change of microglia to various deleterious events is a de-ramification reaction in which the number of endpoints is progressively decreased, and cells eventually becomes an amoeboid morphology [56–58]. This de-ramification reactions are correlated to microglial activation [56]. In our research, we found that COG1410 suppressed morphological change of microglia after CCI. Consistent with previous study, suppression of neuroinflammation by NLRP3 inhibition also attenuated morphological change of microglia (alleviation of microglial endpoints decrease) after experimental SAH in mice [59]. At the same time, we strictly verified that TREM2 could regulate the states of microglia by using TREM2 KO mice. In addition, we clarified that Akt and its downstream CREB/BDNF participated in the protective effects of TREM2 activation and we located this signalling pathway on microglia but not on neurons. Although we found that TREM2 was expressed on both microglia and neurons after CCI, which was consistent with results after ICH by Chen et al., they also found that astrocytes expressed TREM2 after ICH. Intriguingly, BDNF from neurons also changed after CCI, which could be explained by changes in inflammation levels [60]. However, the role of TREM2 expressed by neurons after CCI has not been investigated and needs further research in the future.

Cognitive dysfunction is a common adverse consequence in TBI patients. Most patients with TBI exhibit long-term memory and attention deficits, depression, and fatigue. As mortality rates have declined for severe TBI, attention has focused on the cognitive, affective, and behavioural sequelae of TBI [61–63]. In animal models, learning and memory decline and psychiatric disorders often emerge in the chronic phase after TBI [42, 64]. Our results showed that cognitive deficits and anxiety-like behaviours were present but there was no motor dysfunction at 2 weeks after CCI. Treatment with COG1410 alleviated these deficits. In the beam walking test, all mice traversed the beam with a fast speed at 13 d after CCI, but CCI mice showed more footslips when they crossed the beam. Indeed, balance and postural orientation are serious problems in TBI patients [65]. The results from MEP recording might explain these deficits. COG1410 treatment reduced the footslips but did not reduce the latency of MEP. The mechanism of this phenomenon needs further study. Previous studies have shown that a decrease in theta oscillations in the hippocampus is a marker of TBI [66–68]. In our study, we found that the PSD of theta oscillations in contralateral CA1 decreased at 2 weeks after CCI, and COG1410 increased the PSD of theta oscillations in CA1. A previous study reported that continuous 7.7 Hz theta stimulation of the medial septum significantly increased hippocampal theta oscillations, and improved cognitive function after TBI in mice [69]. Our results further support these findings, indicating that in order to improve cognitive impairment after TBI, we must consider early treatment and chronic functional treatment together. In addition, the open field test and Morris water maze results showed that COG1410-induced TREM2 activation improved neurological functions at 2 weeks after CCI. However, TREM2 knockout abolished this protective effect. Our results robustly verify that TREM2 plays a critical role in neural recovery after TBI.

COG1410, an apoE mimic peptide, has been proven to exert neuroprotective effects in TBI by alleviating neuroinflammation, neural death, and BBB disruption [36, 70, 71]. However, its exact mechanism has not yet been elucidated. In addition, COG1410 has also been reported to have protective effects in experimental subarachnoid haemorrhage and cerebral ischaemia/reperfusion injury [72, 73]. All of these studies show that COG1410 can inhibit microglial activation, neuroinflammation, oxidative stress, and cell death, indicating the potential therapeutic value of COG1410 in acute brain injury. Recent studies have proven that apoE is a novel and high-affinity ligand of TREM2 [25, 26]. Moreover, the Akt/CREB/BDNF signalling pathway was reported to be crucial for neuronal survival after TBI [31]. In addition, this signalling axis plays an essential role in the pathogenesis of neurodegeneration by modulating neuroinflammation and alleviating neural apoptosis and oxidative stress [74]. As we discussed earlier, Akt is one of the important downstream targets of TREM2. Moreover, BDNF has been identified to play a prominent role in the neuronal survival, axonal sprouting, and synaptogenesis after TBI [75]. Overall, we hypothesized that COG1410 exerts neuroprotective effects by activating TREM2 and its downstream signalling pathway. In our study, we found that COG1410 treatment could alleviate neural injury after CCI in mice by suppressing neuroinflammation and promoting neural survival which was consistent with previous studies. However, TREM2 depletion abolished the neuroprotective effects of COG1410 and nullified the effects of COG1410 on downstream signalling of TREM2 in microglia.

Several limitations of this study need to be discussed here. The TREM2 receptor is a major pathology-induced immune signalling hub that senses tissue damage and activates robust immune reactions, which indicates that TREM2 regulates the functions of the body in a sophisticated way. Our study focused only on one of its downstream targets, and further study is needed to explore the other mechanisms underlying the neuroprotective effects of TREM2 after TBI. In the present study, two simple protein markers CD86 and CD206 were double-stained with Iba1 on microglia, and we found that TREM2 could regulate the states of microglia. Although many studies show that CD86^+^ and CD206^+^ microglia exert harmful and protective effects, respectively [76, 77], it is now acknowledged that microglial response is highly dynamic and complex and that M1 and M2 like classification is an oversimplification and not biologically relevant [78]. In the future, we will study microglia with multidimensional ways by integration of epigenetic, transcriptomic, metabolomics and proteomic data. In addition, we found that TREM2 was also expressed on neurons after CCI. Although we demonstrated that the Akt/CREB/BDNF pathway did not take place in neurons, it is necessary to investigate the roles of TREM2 in neurons after TBI. In addition, we did not investigate whether the activation of CREB was regulated by Akt alone or together with other signalling pathways. Finally, the roles of BDNF in the neuroprotective effects of TREM2 need to be studied in more detail. Therefore, in future studies, we will further investigate the neuroprotective effects of TREM2 after TBI.

## Conclusions

Overall, we identified that TREM2 activation by COG1410 alleviated neural damage through activation of the Akt/CREB/BDNF signalling axis in microglia. Finally, COG1410 treatment improved neurological behaviours and brain electrophysiological activity after TBI. Therefore, activation of TREM2 may be a potential therapeutic strategy for the management of TBI patients.

## Supplementary Information

**Additional file 1**. Additional methods and Additional Figures S1–S9.

## Data Availability

The authors declare that all supporting data are available within the article and the supplemental data or obtained under reasonable requirements.
