# Supplementary material for: TREM2 activation alleviates neural damage via Akt/CREB/BDNF signalling after traumatic brain injury in mice
Source: J Neuroinflammation. 2022 Dec 3;19:289. doi: 10.1186/s12974-022-02651-3 (PMC9719652; doi:10.1186/s12974-022-02651-3)
Supplement: Supplementary file 1 — Additional file 1. Additional methods and Additional Figures S1–S9. [file 12974_2022_2651_MOESM1_ESM.docx]

**Additional file 1**

**TREM2 activation alleviates neural damage via Akt/CREB/BDNF signaling after traumatic brain injury in mice**

**Additional methods**

**Additional Figures and Figure Legends**

**Additional methods**

**TREM2 genotype identification in F0 and F1 generation mice by polymerase chain reaction (PCR)**

We purchased one male and one female homozygote TREM2 KO mouse from Jackson Lab as F0 generation. And they produced six offspring, which were F1 generation. We used PCR to confirm the TREM2 genotype of the F0 and F1 generation. Briefly speaking, tails of these eight mice and another wildtype (WT) mouse were collected to analysis. The WT mouse was used to as control in TREM2 genotype identification. Total DNA was extracted from tails of mice using an Animal Genomic DNA Quick Extraction Kit for PCR Analysis (Cat# D0065S, Beyotime Biotechnology, Shanghai, China) according to the manufacturer’s instruction. PCR systems were set up in 20 µl volumes and PCR was performed on a PCR System (Bio-Rad, USA). The 20 µl volumes PCR systems included 8 µl ddH_2_O, 10 µl 2 × Taq Master Mix (Cat# P112-AA, Vazyme), 0.5 µl forward primer (10 pmol/µl), 0.5 µl reverse primer (10 pmol/µl), and 1 µl genomic DNA. The program included 5 min at 95 °C; 10 cycles of 30 s at 95 °C ,30 s at 60 °C, and 1 kb/min at 68 °C; 26 cycles of 30 s at 95 °C ,30 s at 55 °C, and 1 kb/min at 72 °C; 5 min at 72 °C; 2 min at 10 °C. Then, the concentration of agarose gel was 2% (Cat# 1110GR100, BioFroxx) in agarose gel electrophoresis. The DNA was dyed by nucleic acid dye (GelstainRedTM, Cat# S2009L, UE). Quantity of base pair was instructed by DNA marker (Cat# BM411-02, Trans DNA Marker Ⅱ). The sequences of the primers for TREM2 mutant genotype as follows: forward sequence, 5’-TTACACAAGACTGGAGCCCTGAGGA-3’; reverse sequence, 5’-TCTGACCACAGGTGTTCCCG-3’. The sequences of the primers for WT genotype as follows: forward sequence, 5’-CCCTAGGAATTCCTGGATTCTCCC-3’; reverse sequence, 5’-TCTGACCACAGGTGTTCCCG-3’. Results are shown in Suppl Fig. 1.

**Additional Figures and Figure Legends**


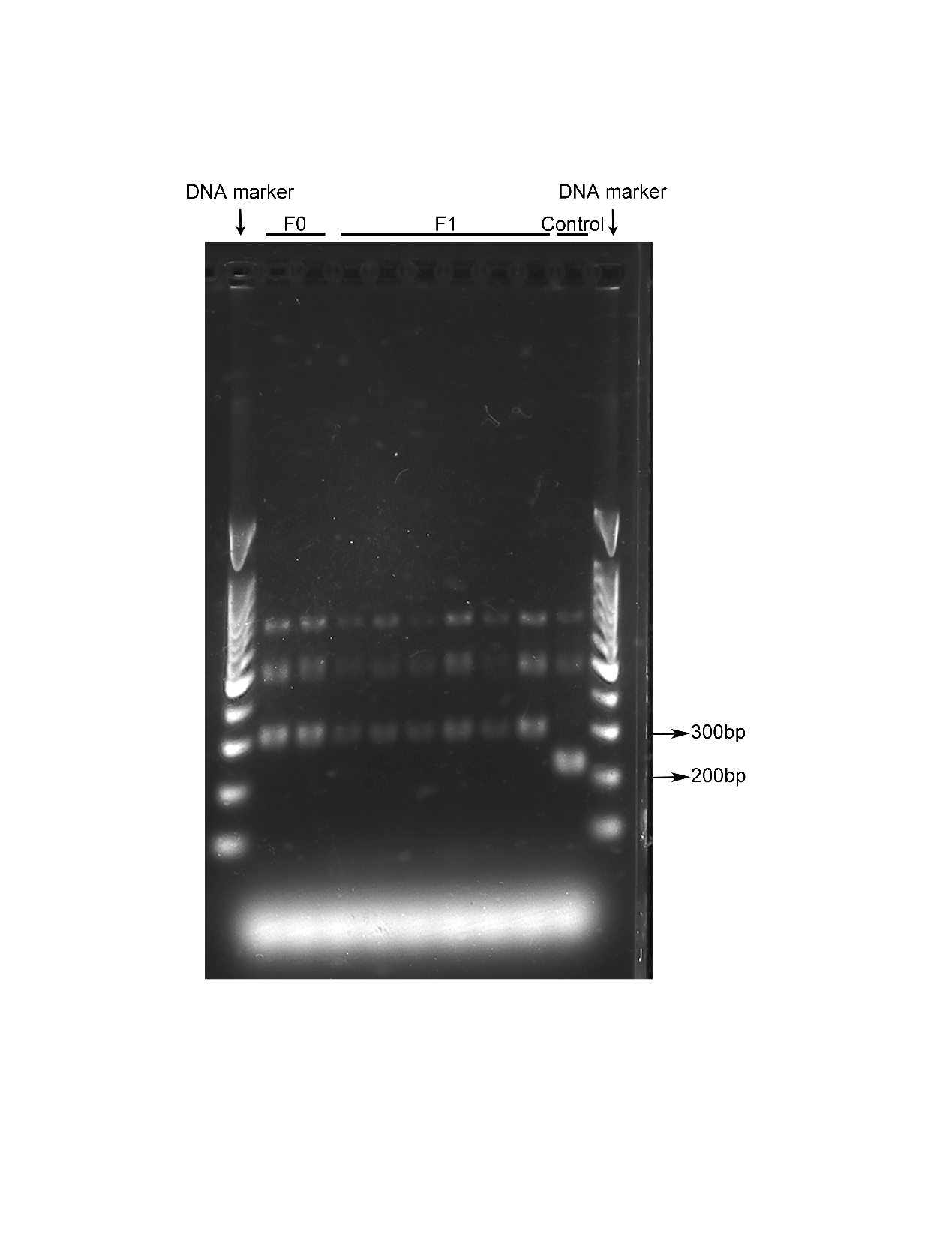


**Fig. S1. Image of TREM2 genotype identification.** The mutant band is localized at approximate 300 bp and the WT band is localized at approximate 200 bp as shown in the image. Consequently, the genotype of F0 and F1 mice were all homozygote TREM2 KO. Adult male TREM2 KO mice used in this study were offspring of the F1 generation here. Besides, the western blot analysis showed that TREM2 was totally abolished in TREM2 KO mice (see text Fig. 9D).


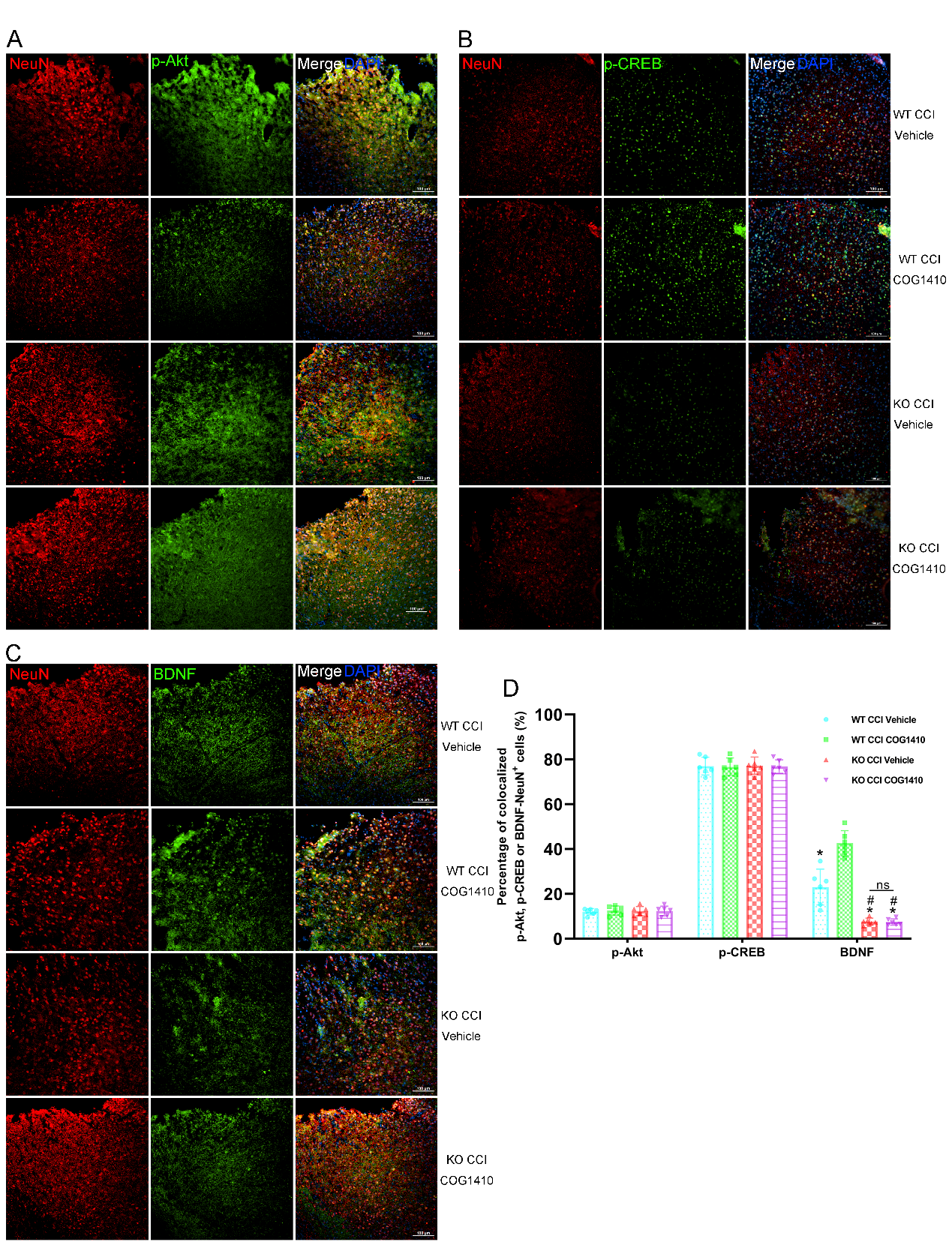


**Fig. S2. Activation of the Akt/CREB/BDNF signaling axis did not occur in neurons A.** Representative images of neurons (NeuN, red) and p-Akt^+^ cells (p-Akt, green) surrounding the lesion sites at 3 d after CCI. Nuclei were stained with DAPI (blue). Scale bar = 100 μm. **B.** Representative images of neurons (NeuN, red) and p-CREB^+^ cells (p-CREB, green) surrounding the lesion sites at 3 d after CCI. Nuclei were stained with DAPI (blue). Scale bar = 100 μm. **C.** Representative images of neurons (NeuN, red) and BDNF^+^ cells (BDNF, green) surrounding the lesion sites at 3 d after CCI. Nuclei were stained with DAPI (blue). Scale bar = 100 μm. **D.** Quantitative analysis of the percentage of colocalized p-Akt-NeuN^+^ cells, p-CREB-NeuN^+^ cells, and BDNF-NeuN^+^ cells. * P<0.05 vs. WT CCI + COG1410; # p<0.05 vs. WT CCI + Vehicle; ns p>0.05 KO CCI + Vehicle vs. KO CCI + COG1410, n=6 per group


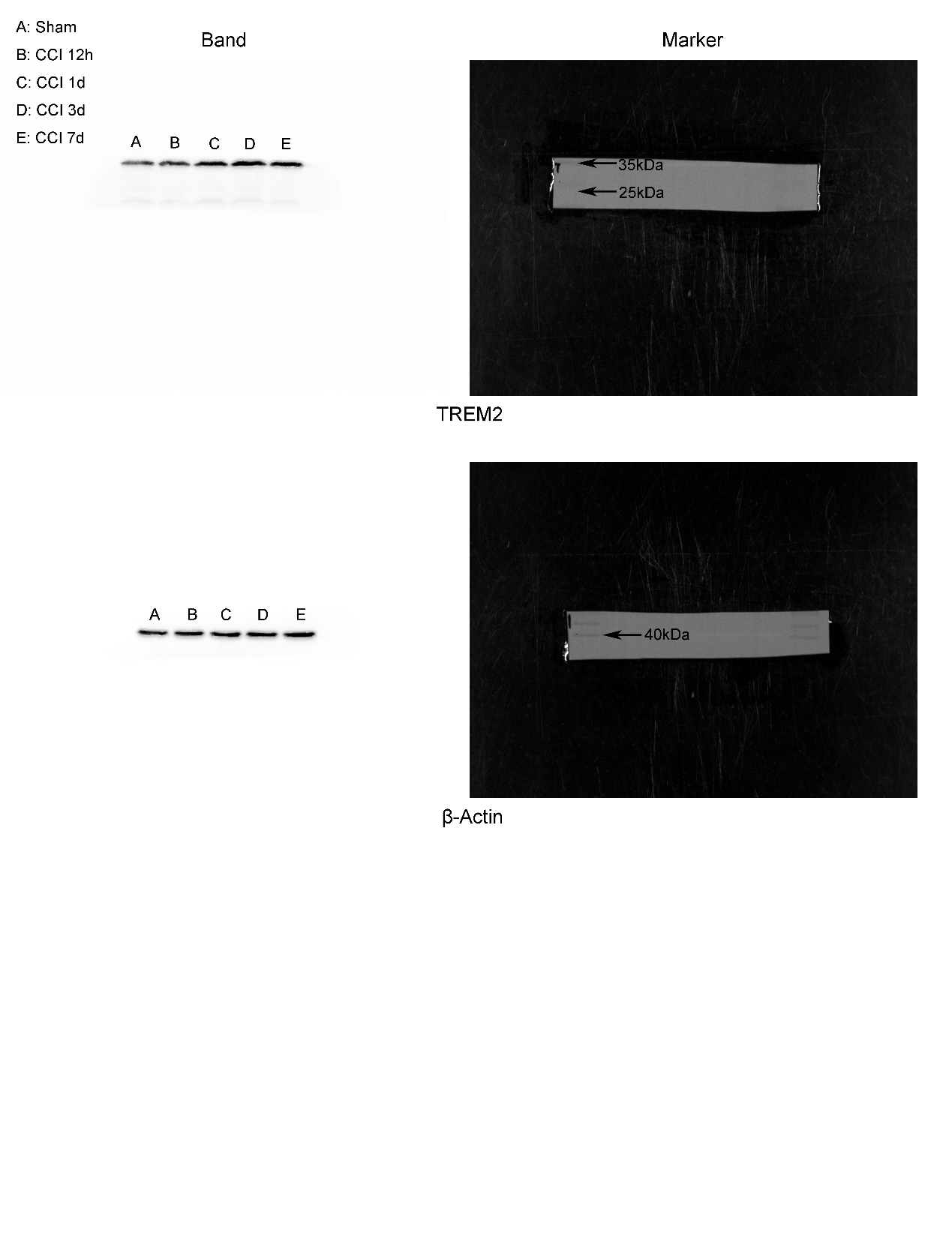


**Fig. S3. Raw western blot bands in Fig. 2A**


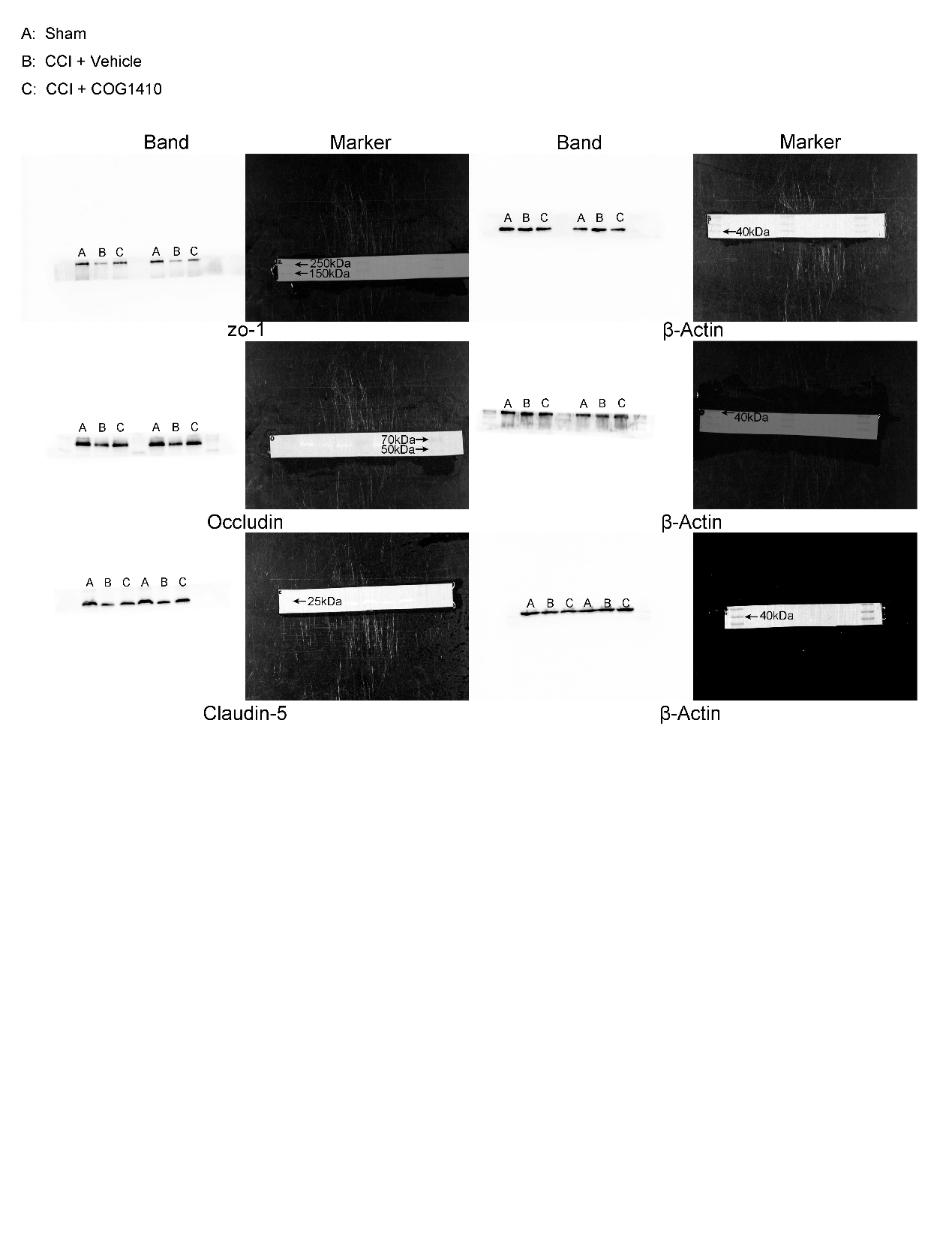


**Fig. S4. Raw western blot bands in Fig. 4F**


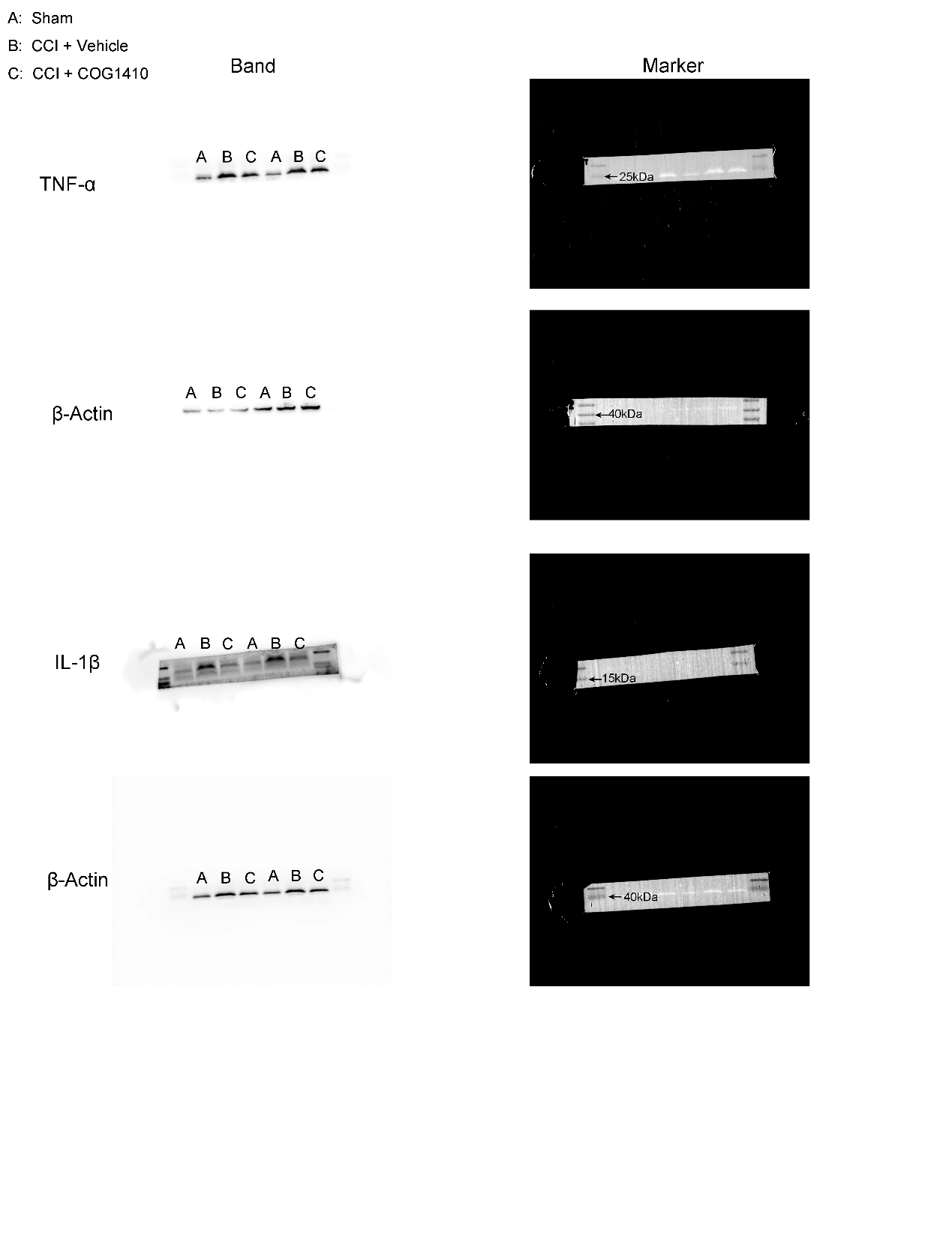


**Fig. S5. Raw western blot bands in Fig. 5J**

**
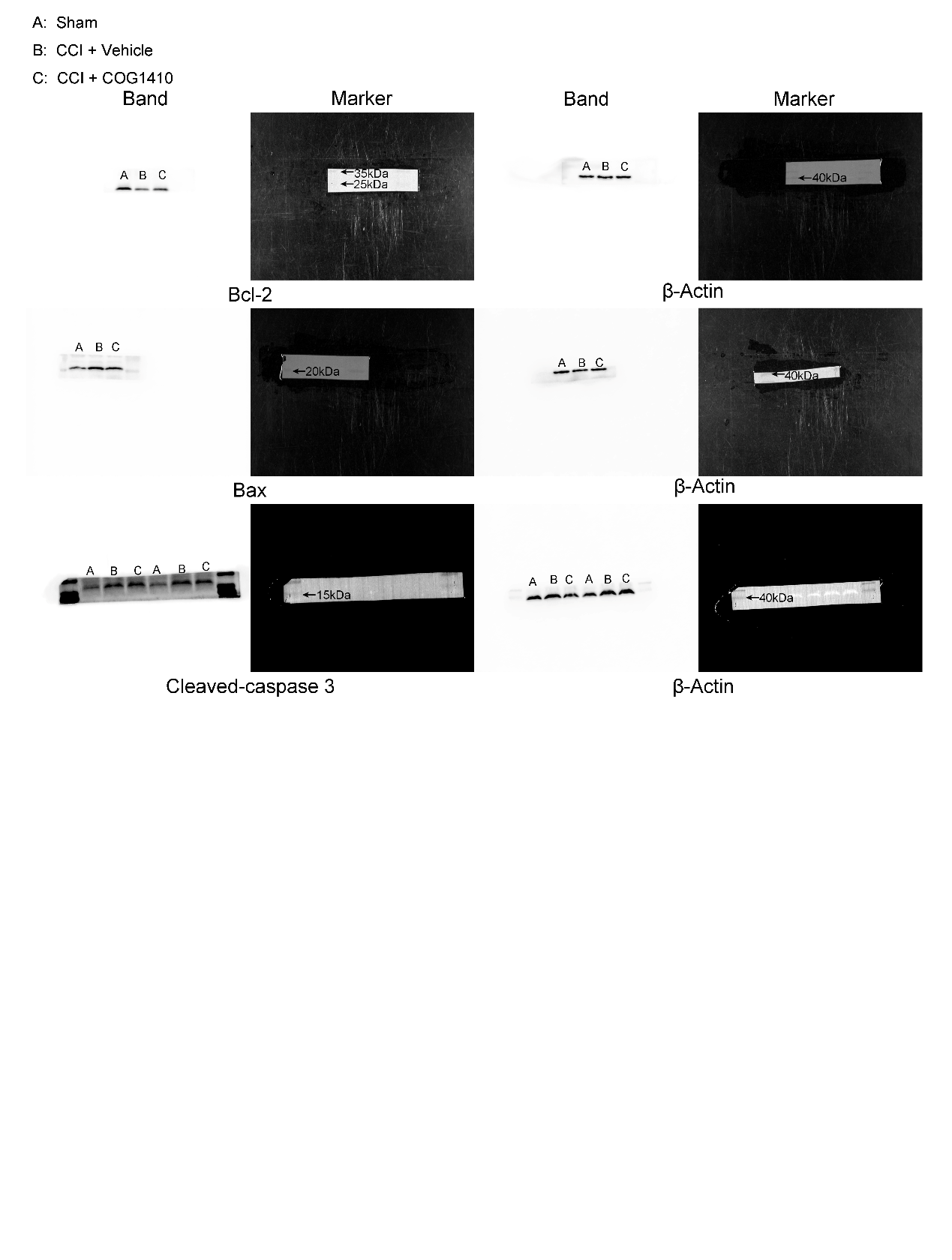
**

**Fig. S6. Raw western blot bands in Fig. 6C**

**
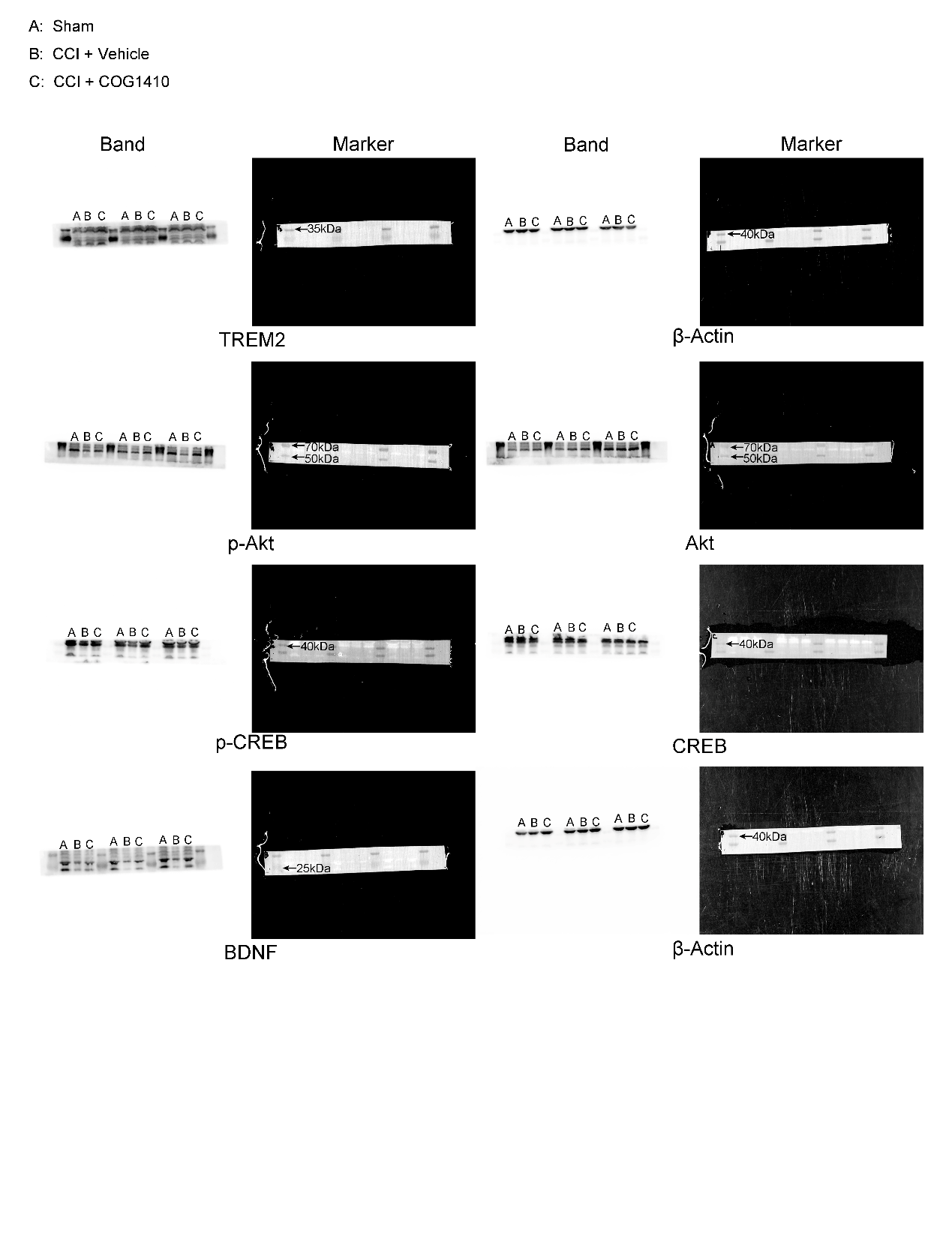
**

**Fig. S7. Raw western blot bands in Fig. 7C**

**
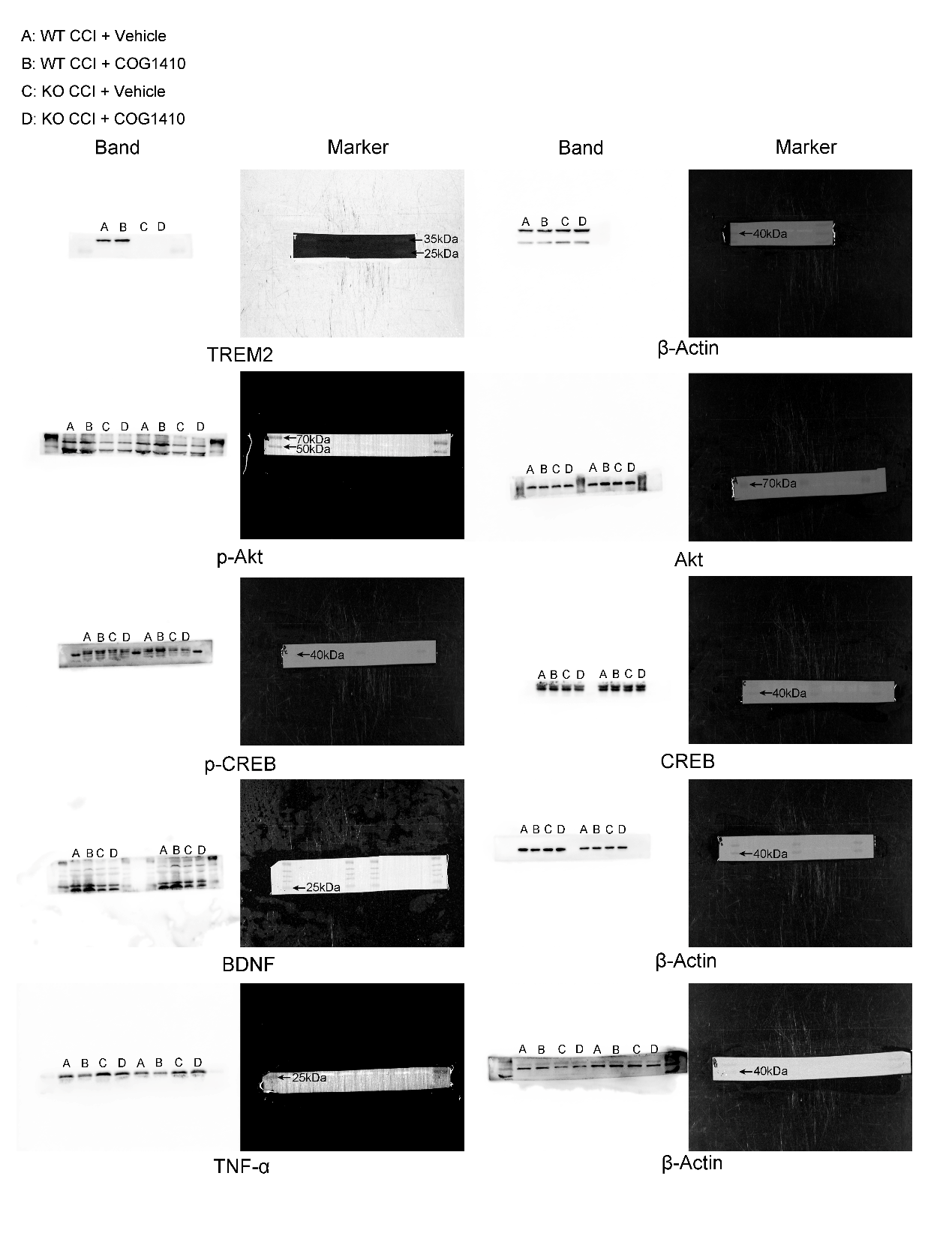
**

**
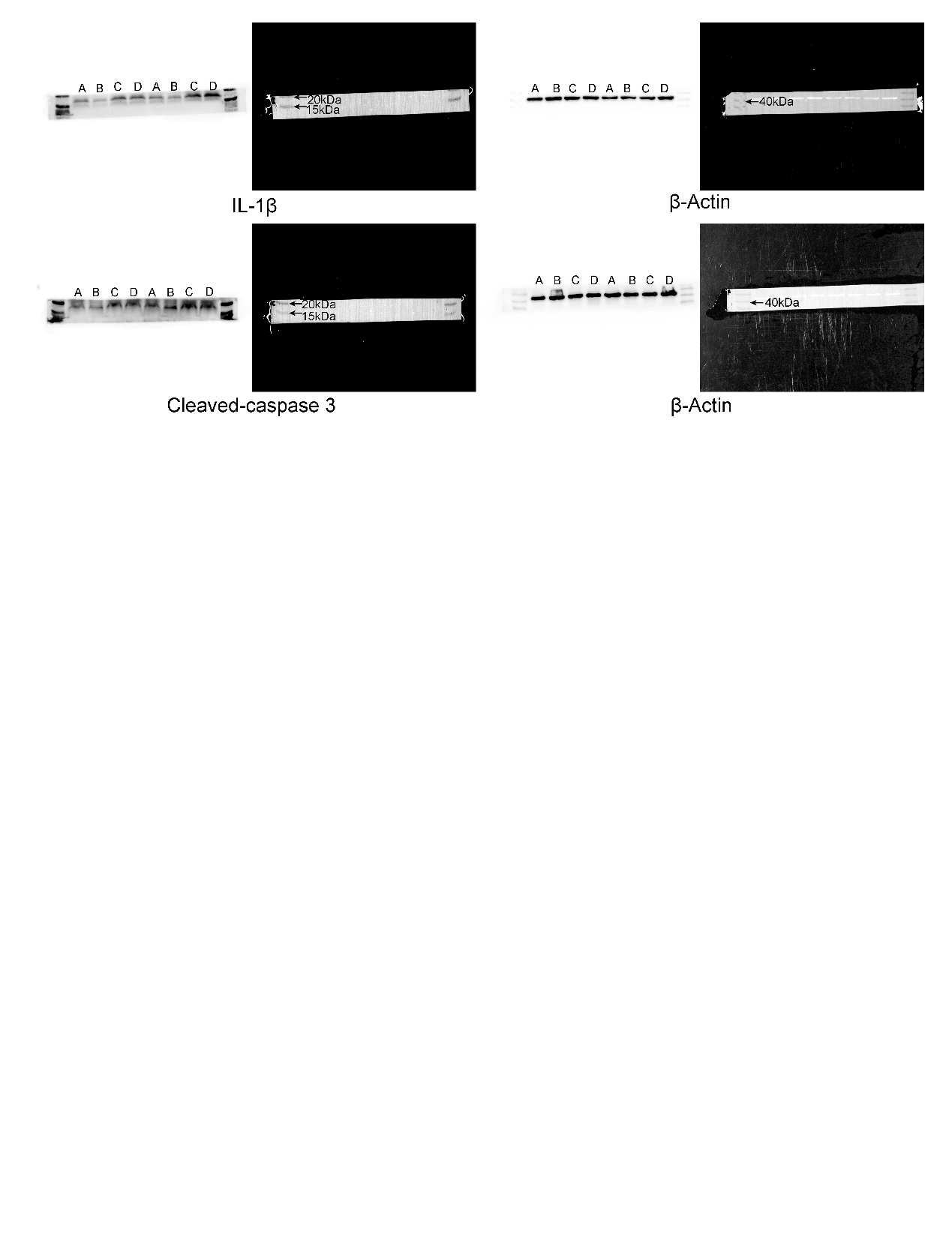
**

**Fig. S8. Raw western blot bands in Fig. 8D**

**
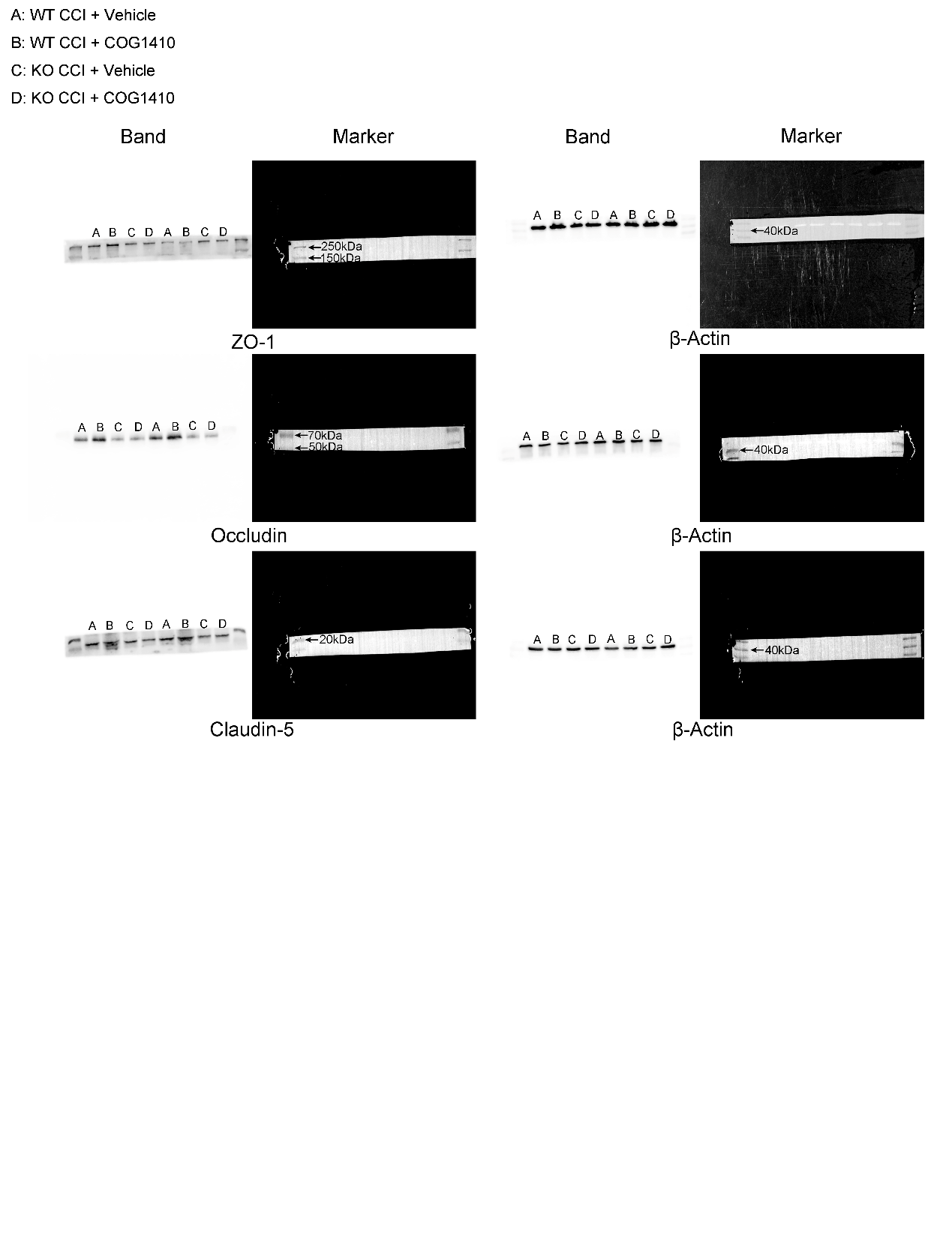
**

**Fig. S9. Raw western blot bands in Fig. 10C**
